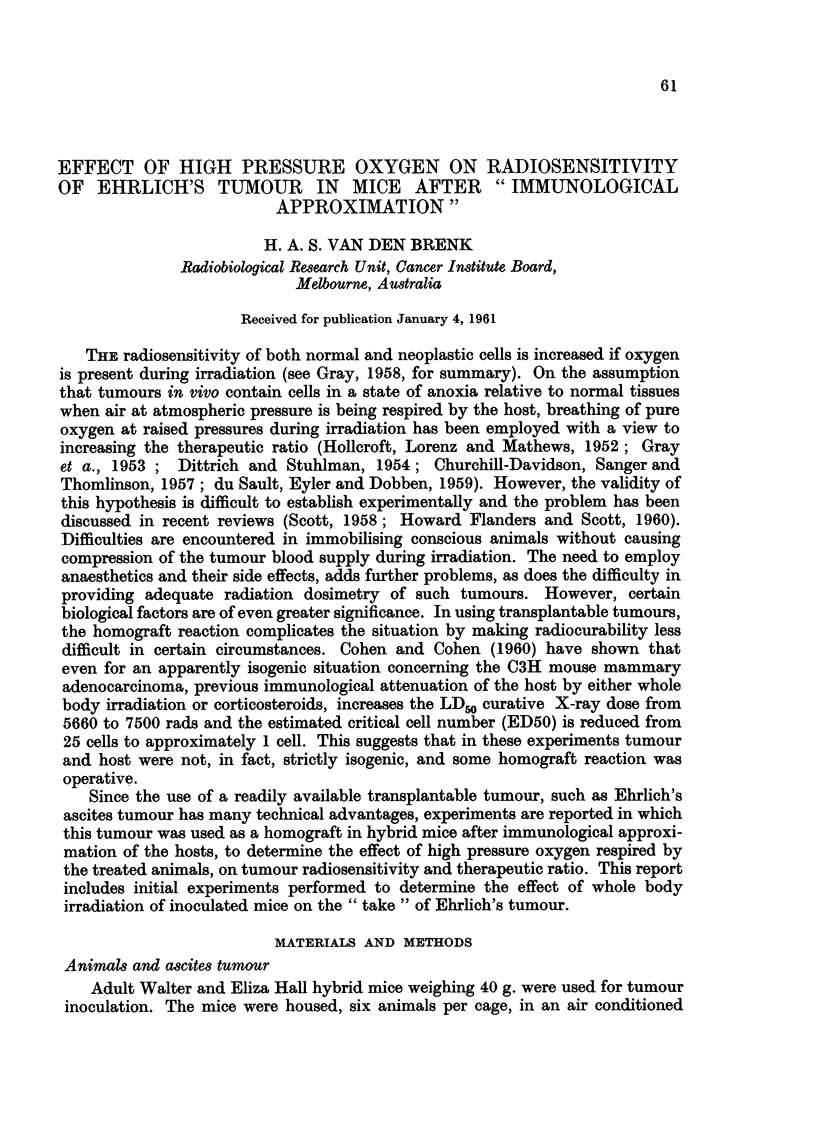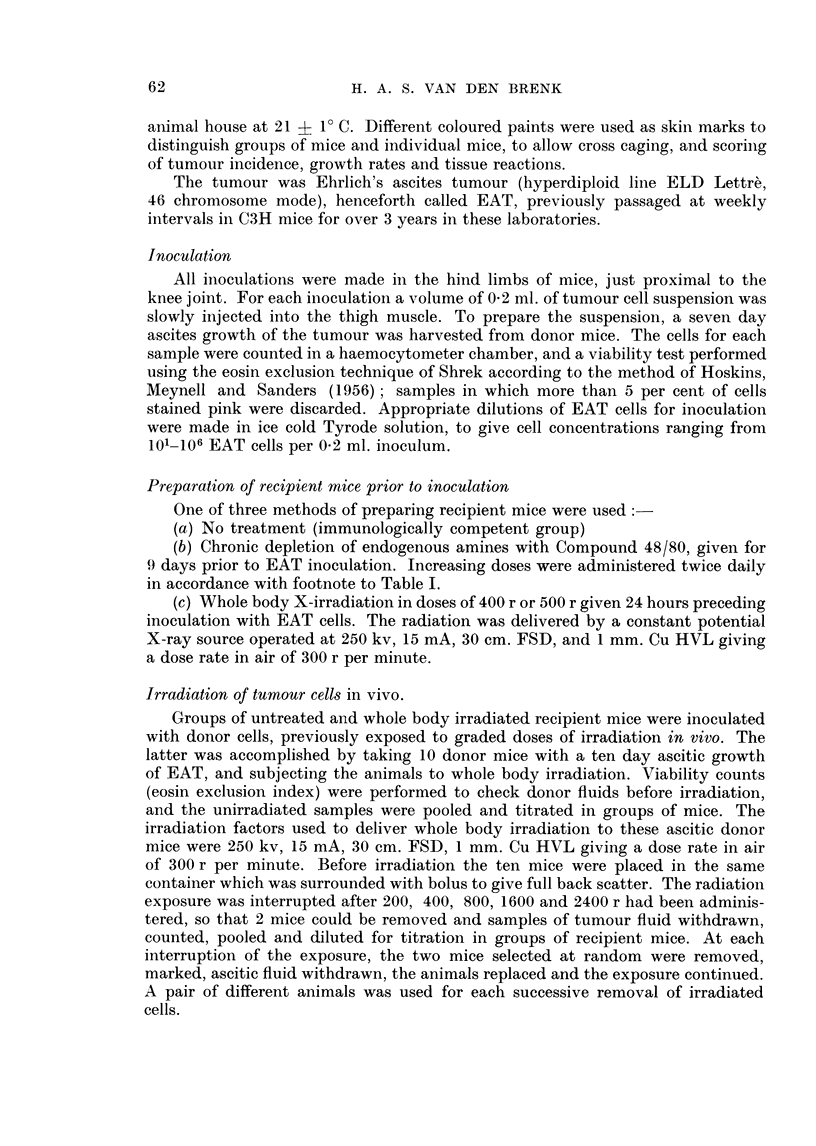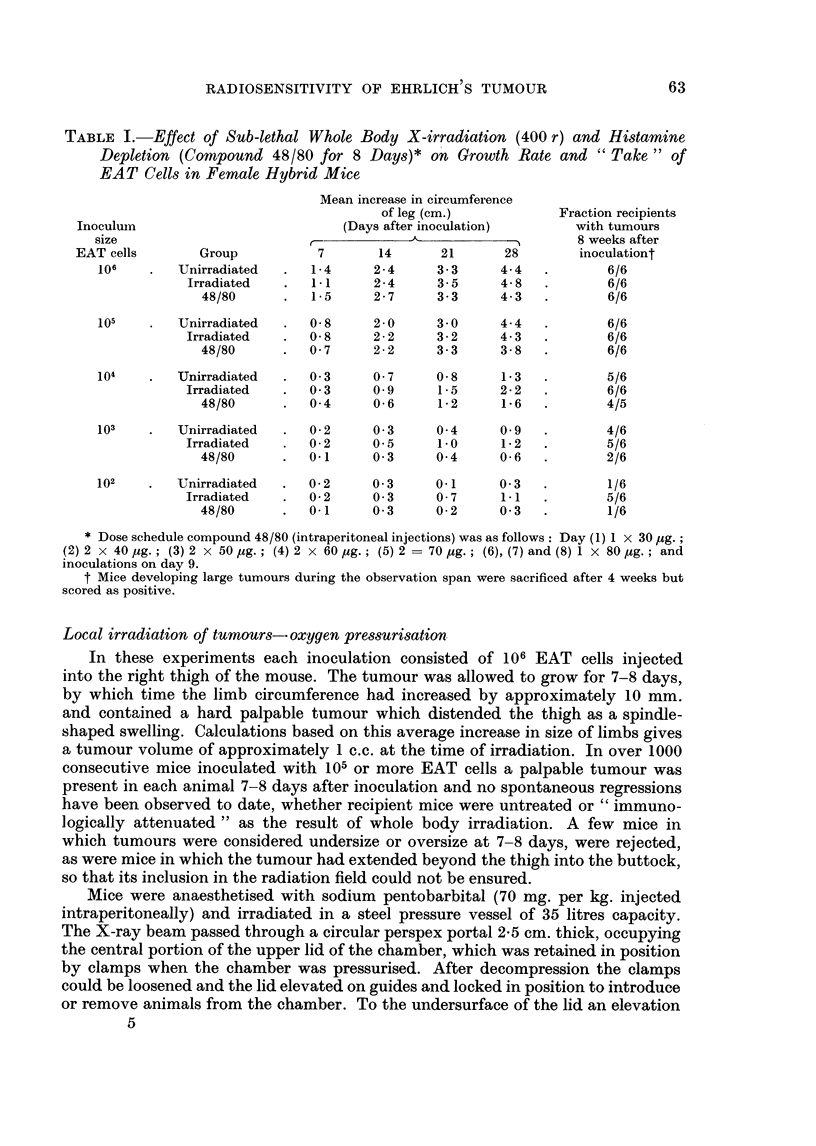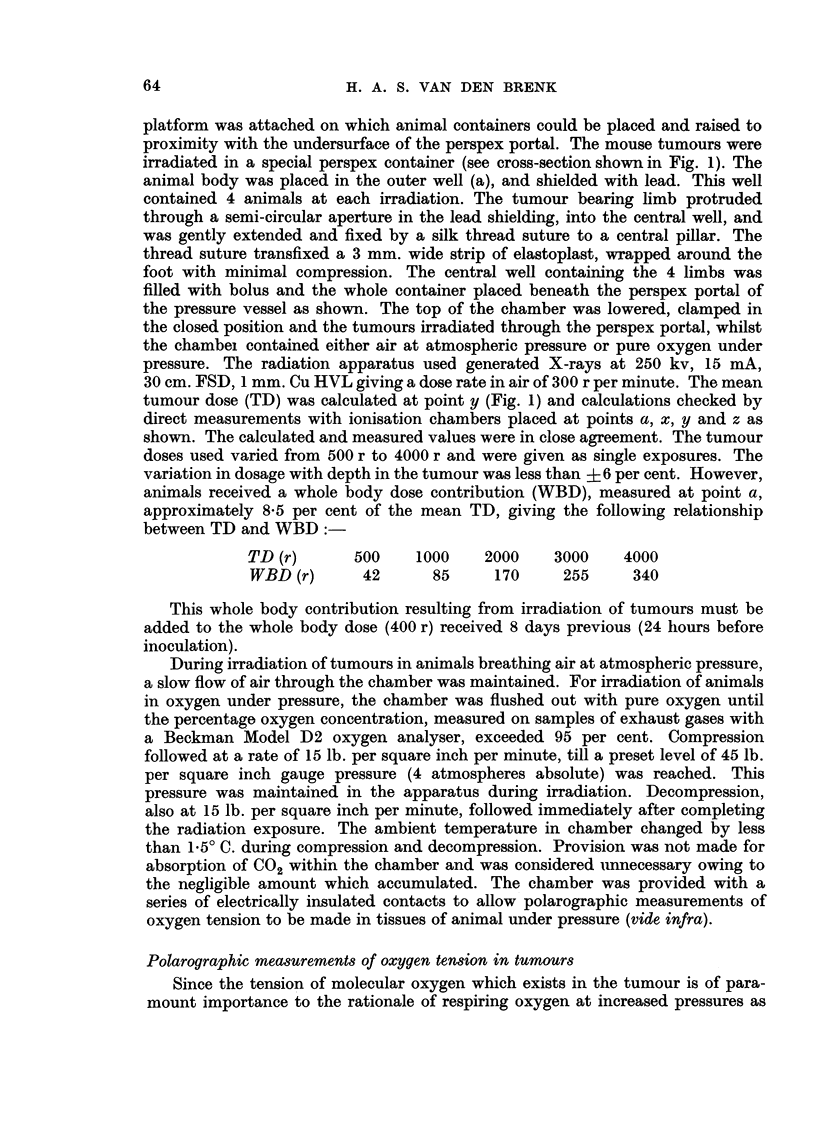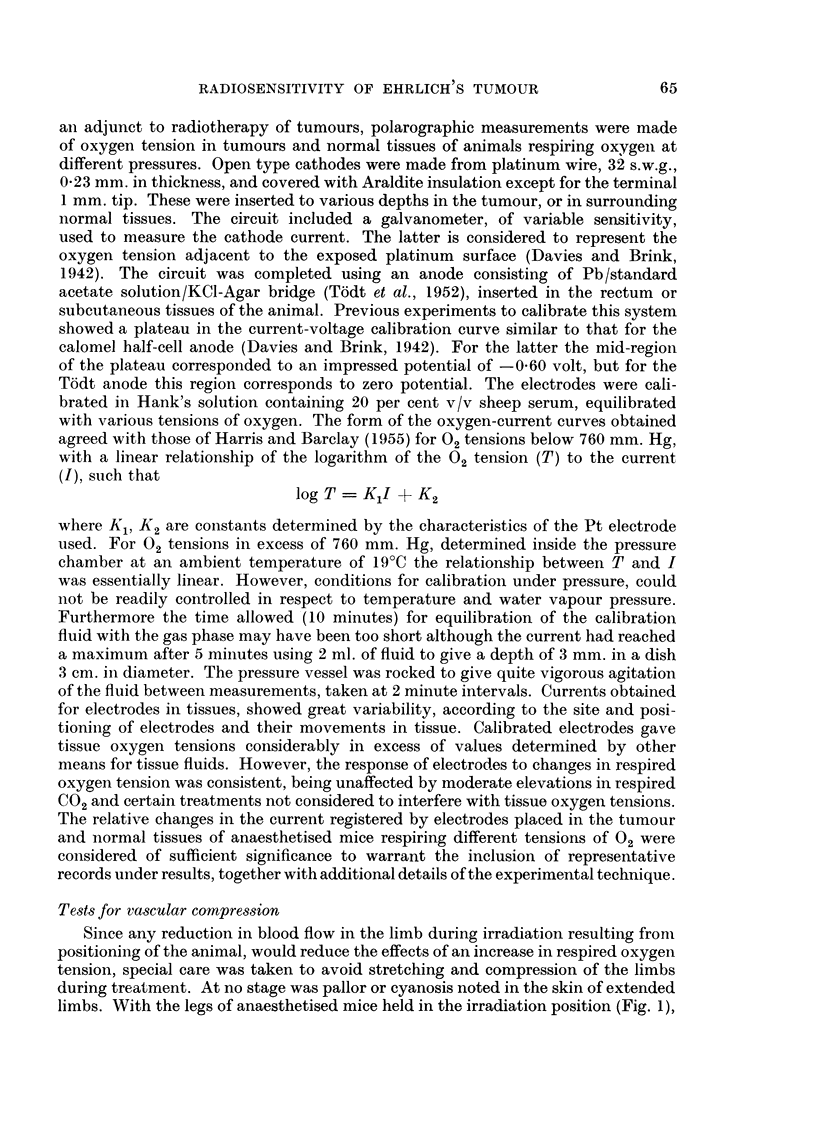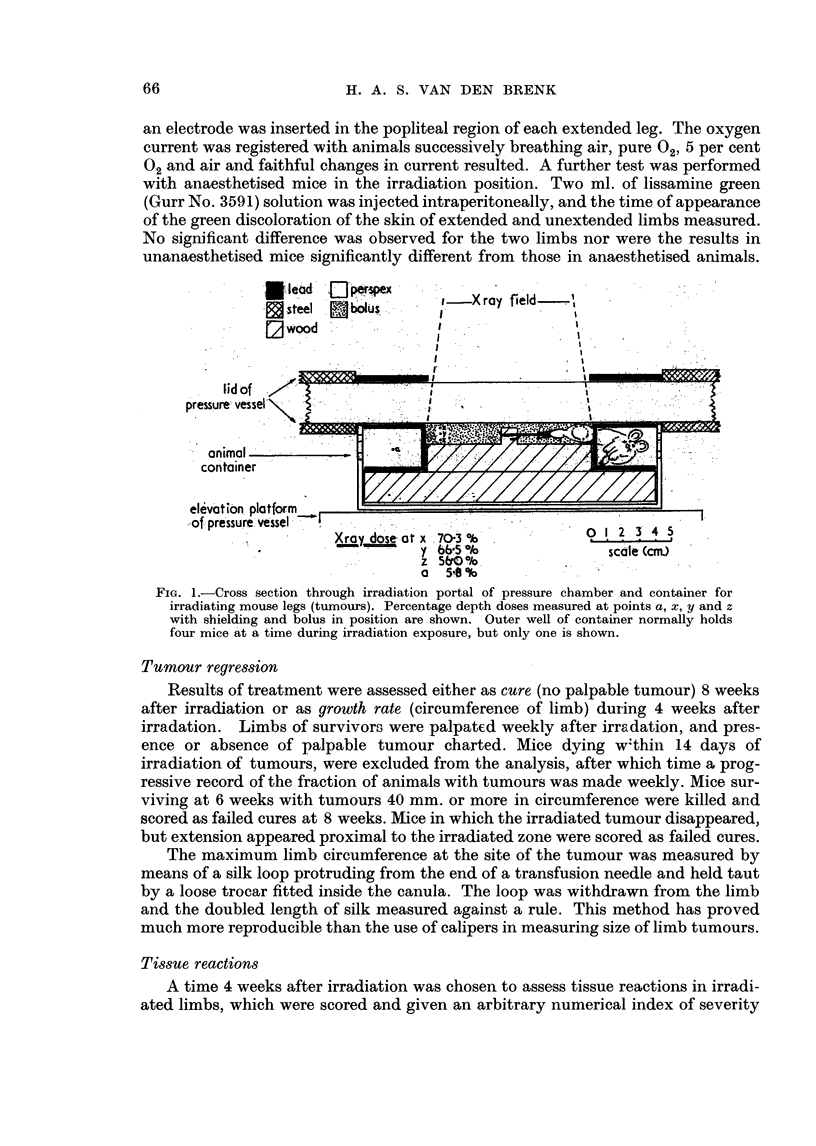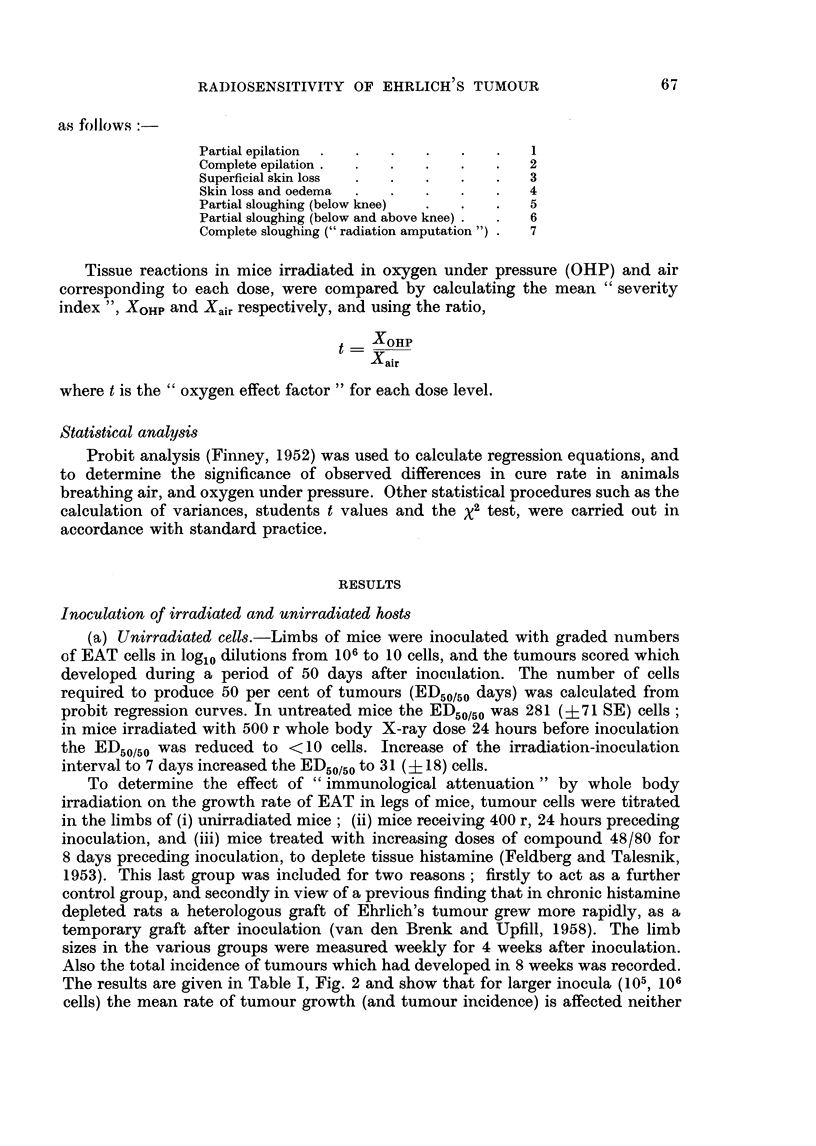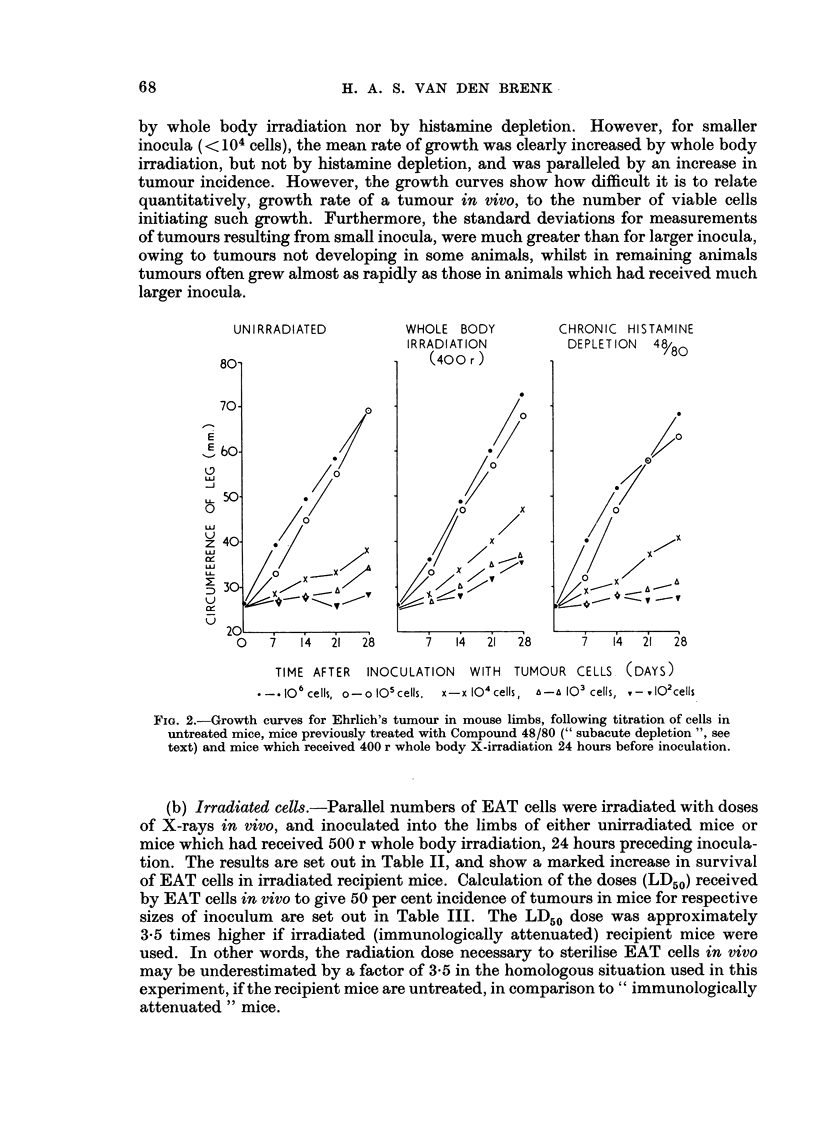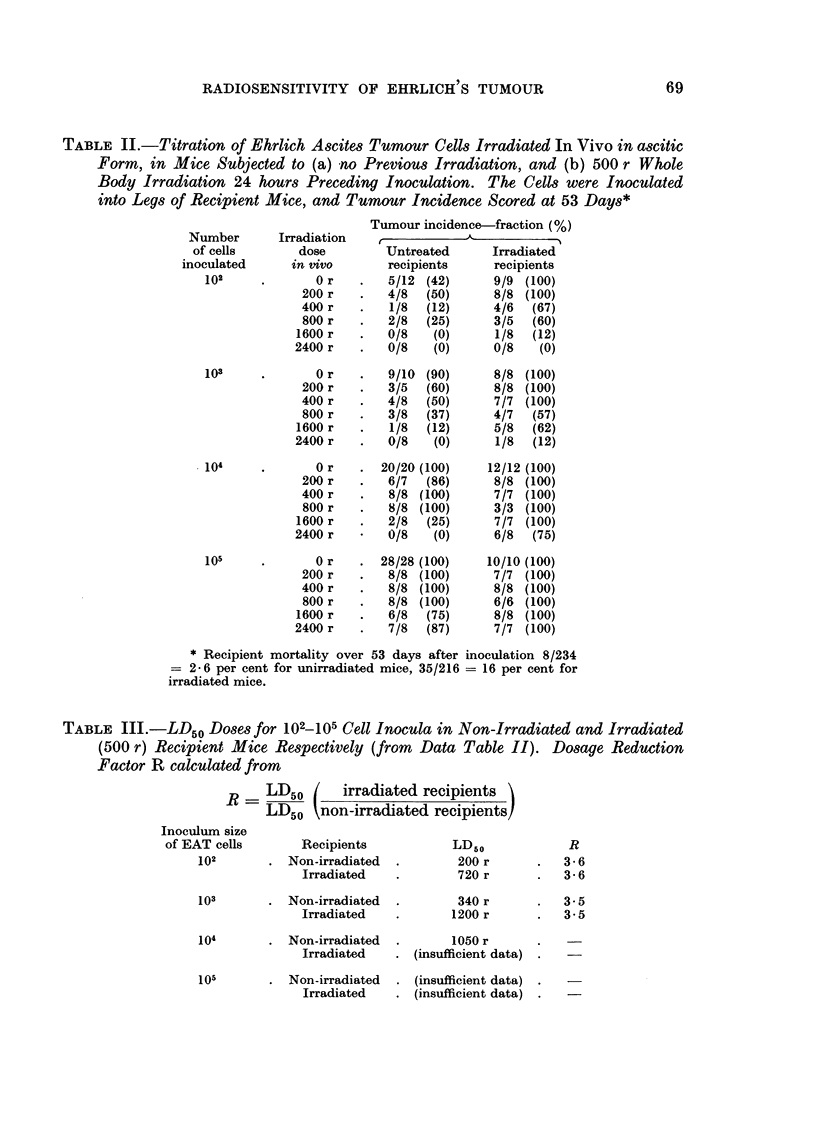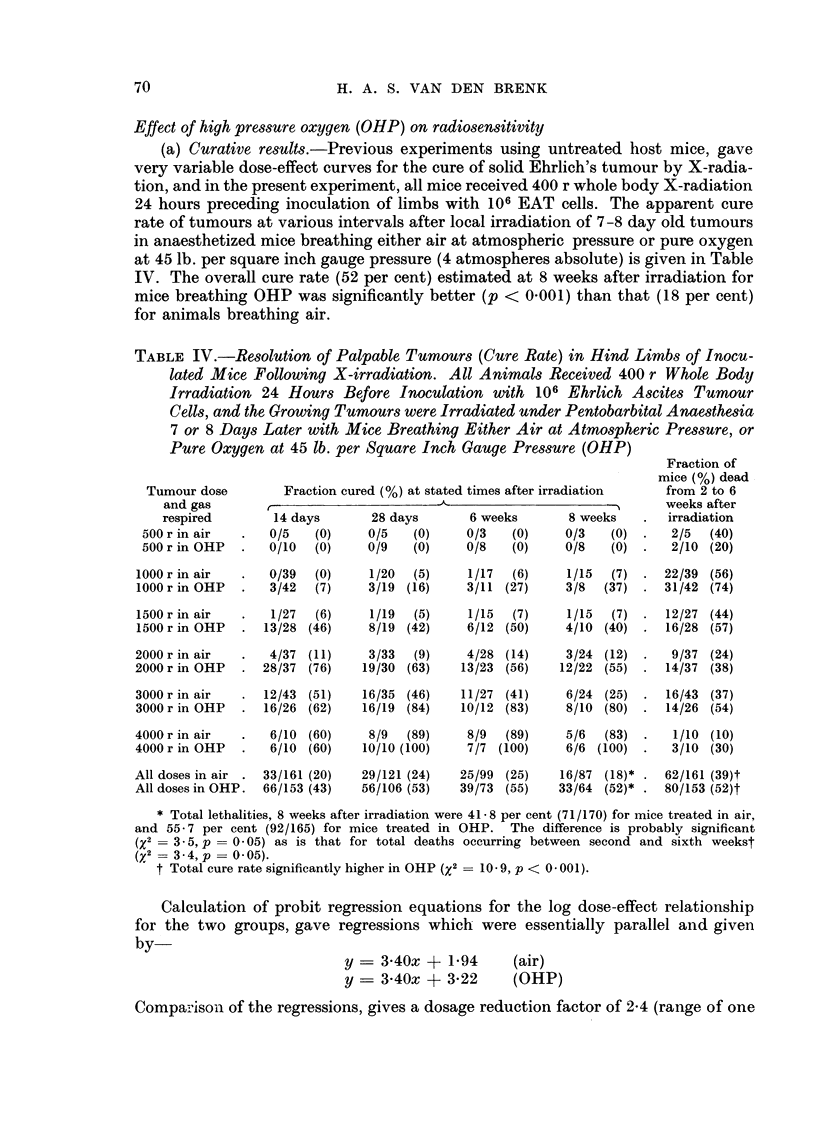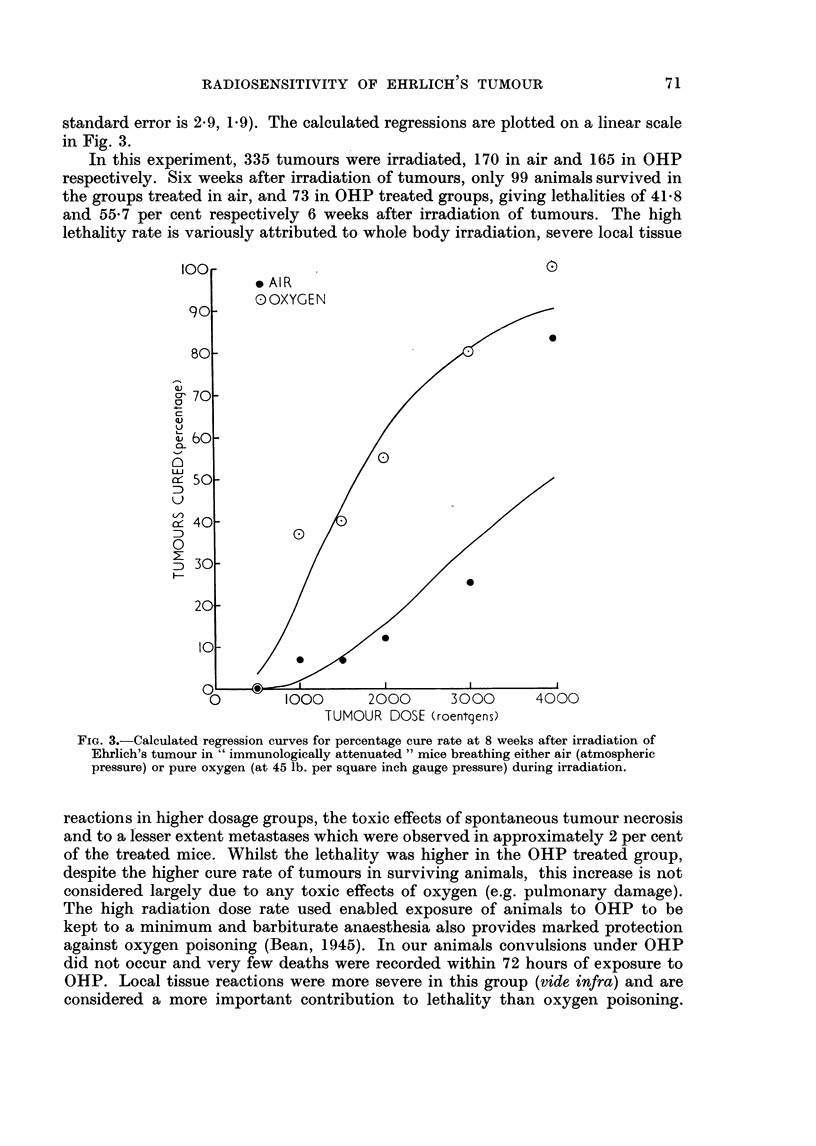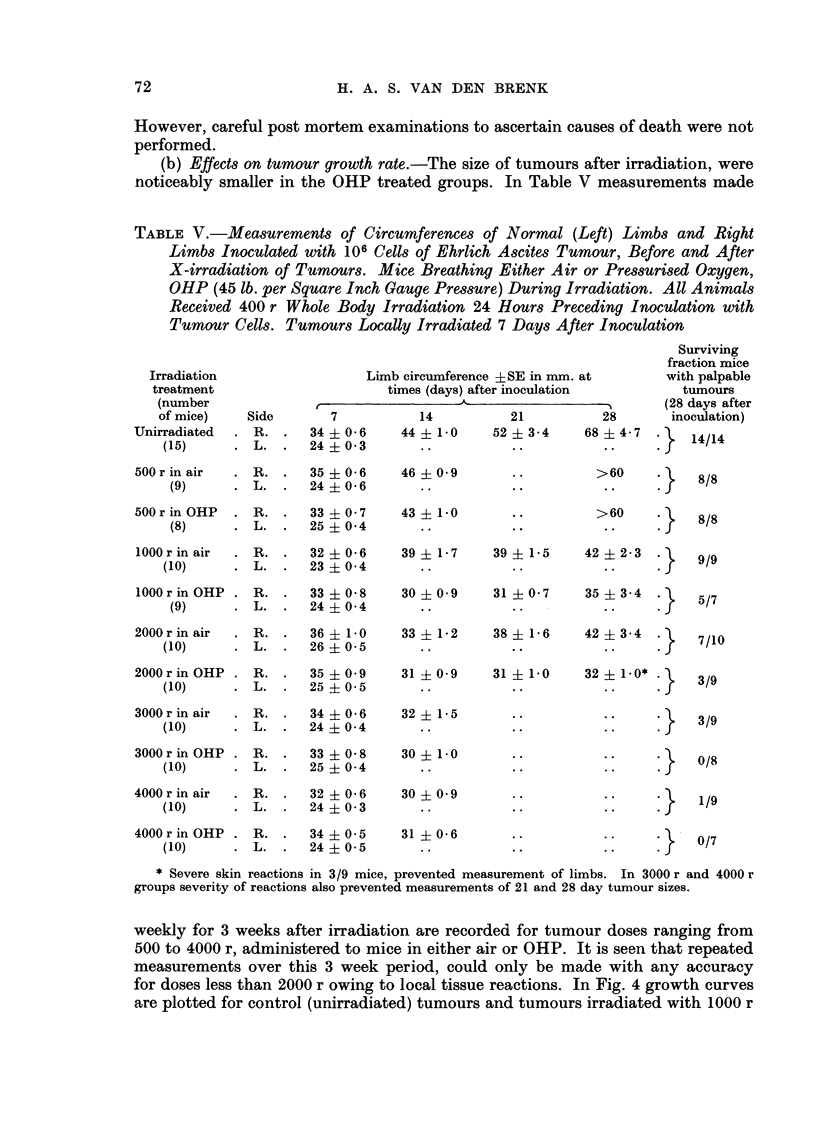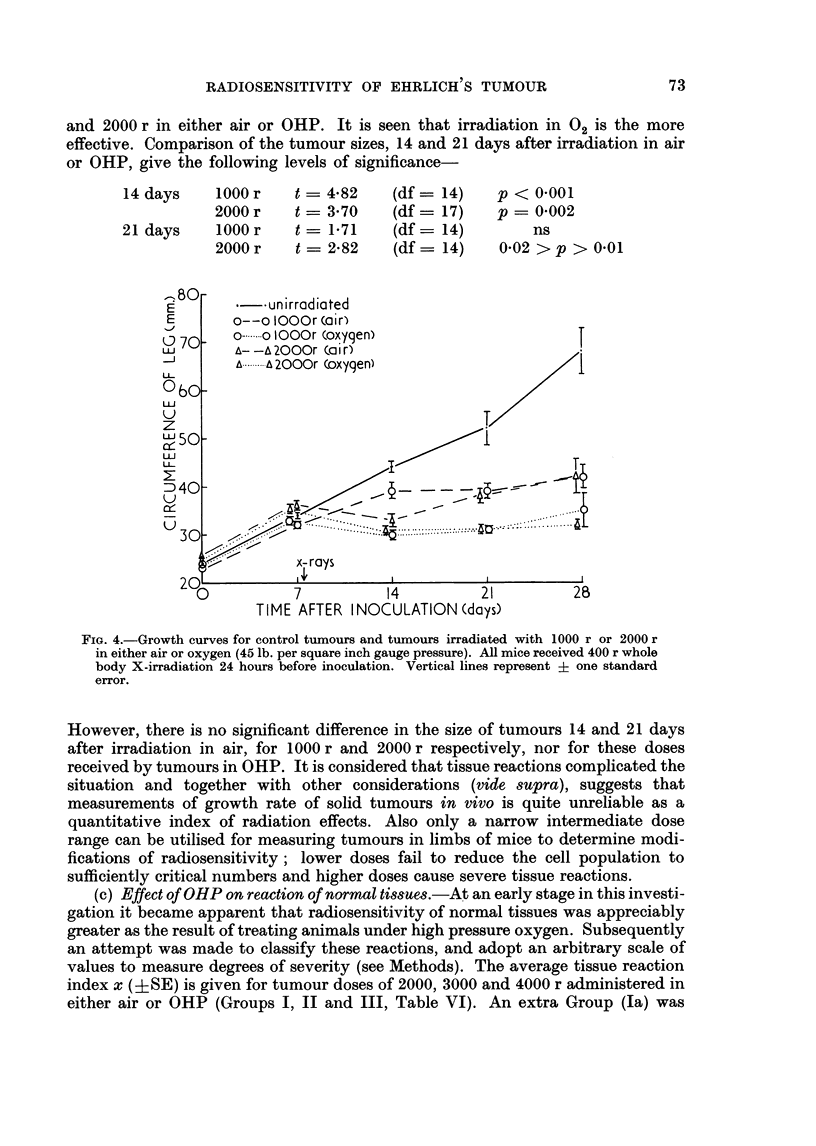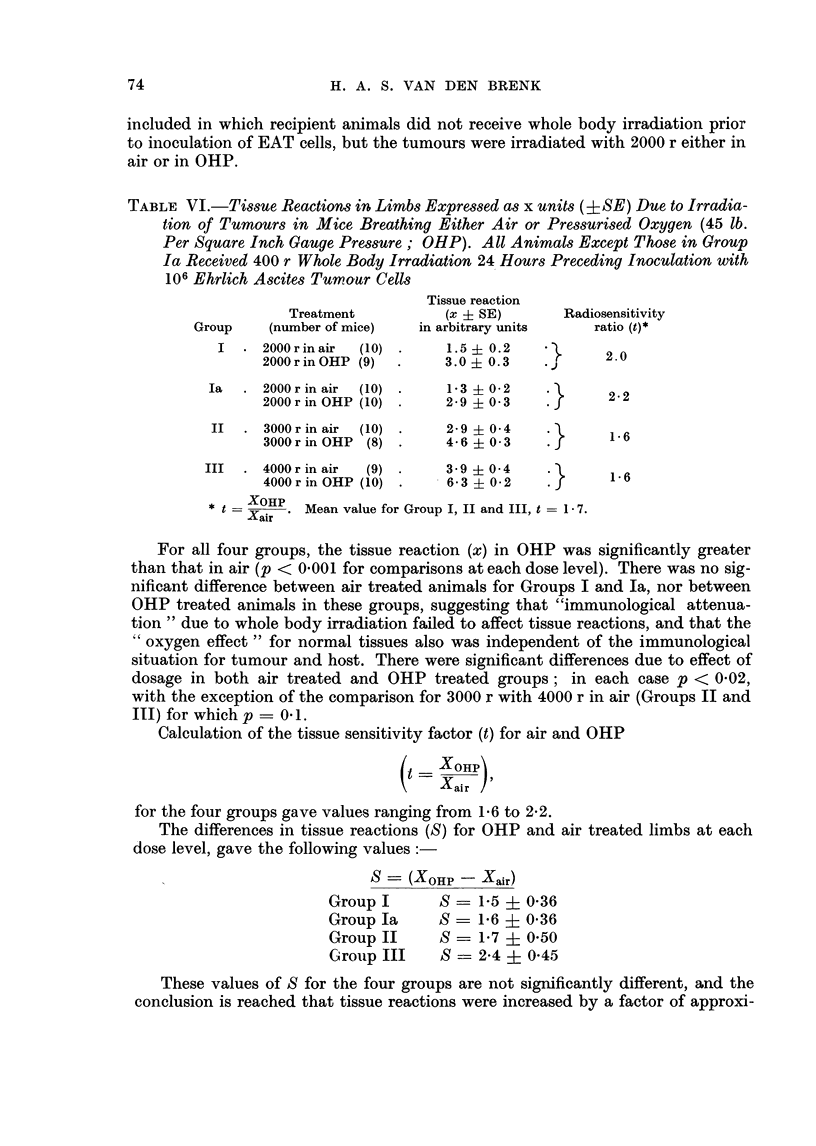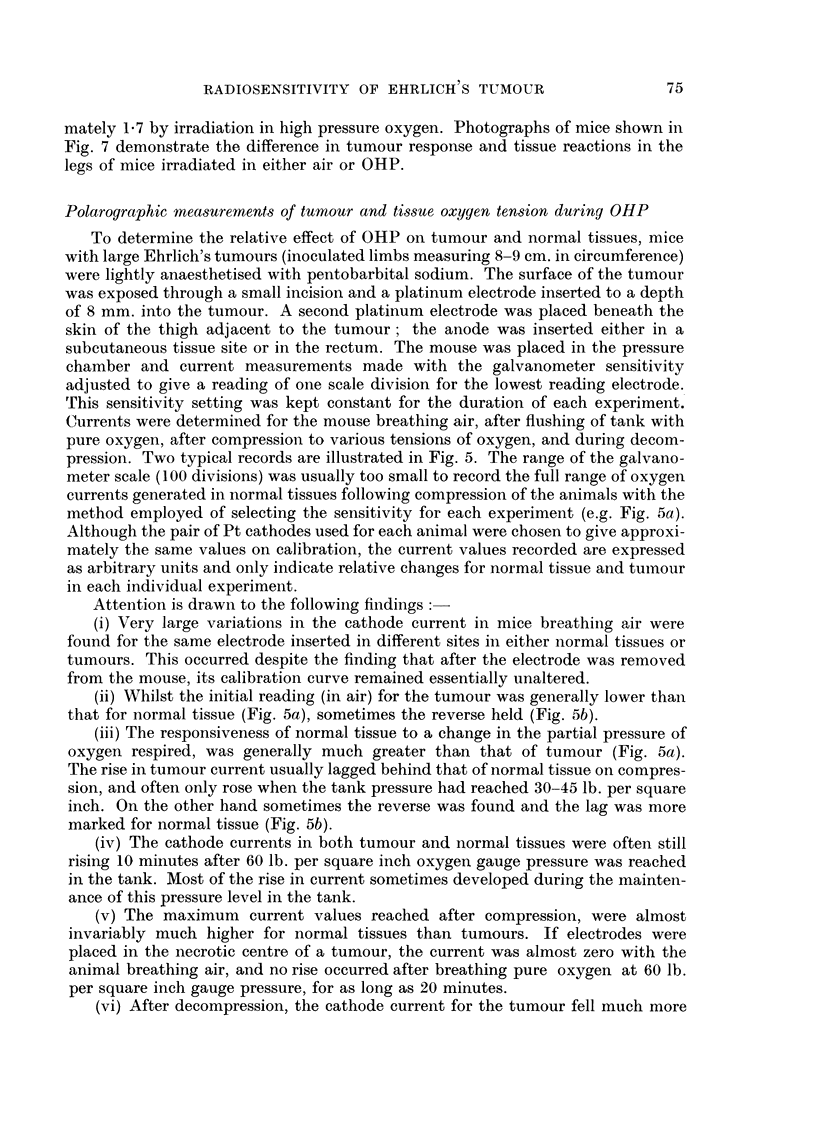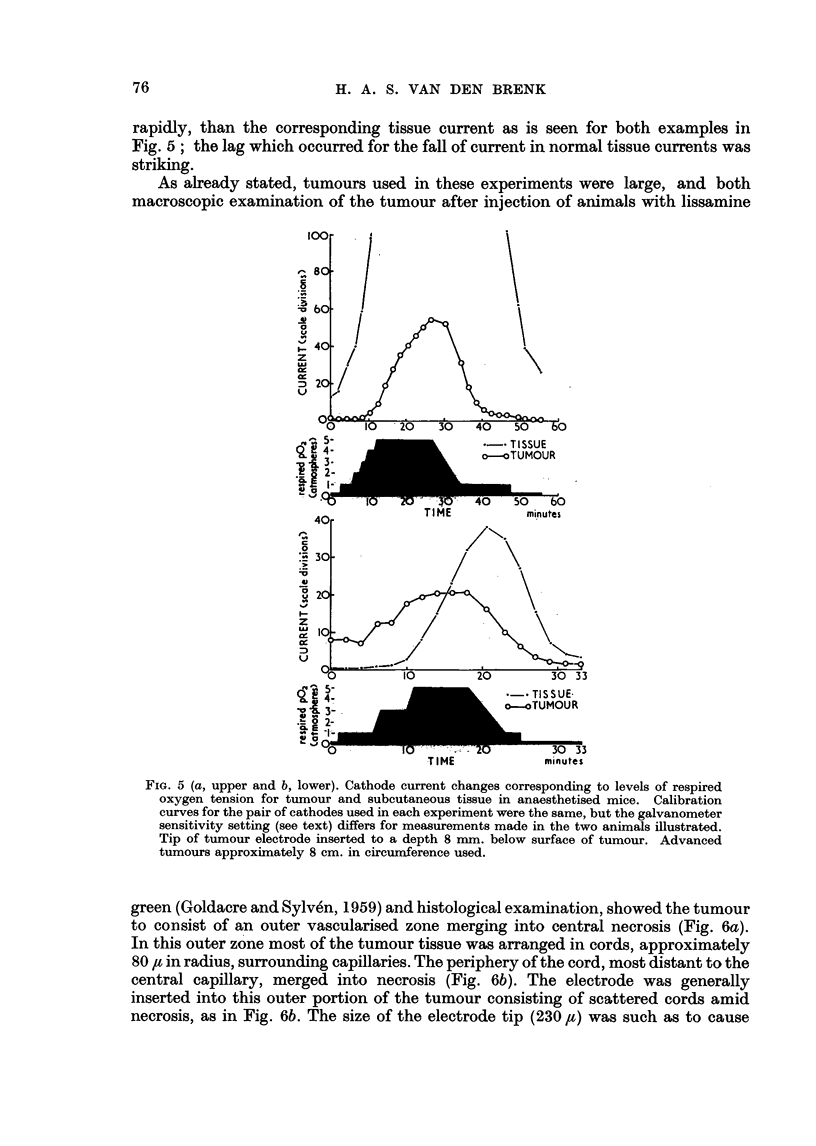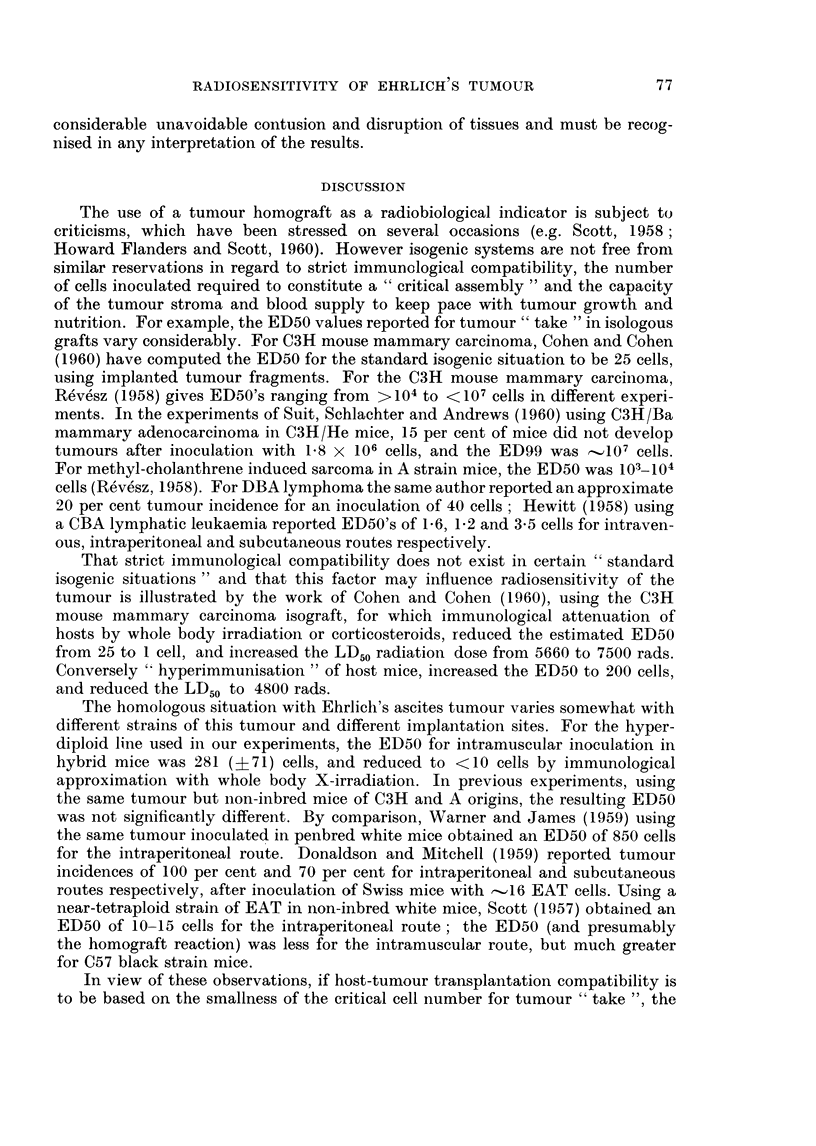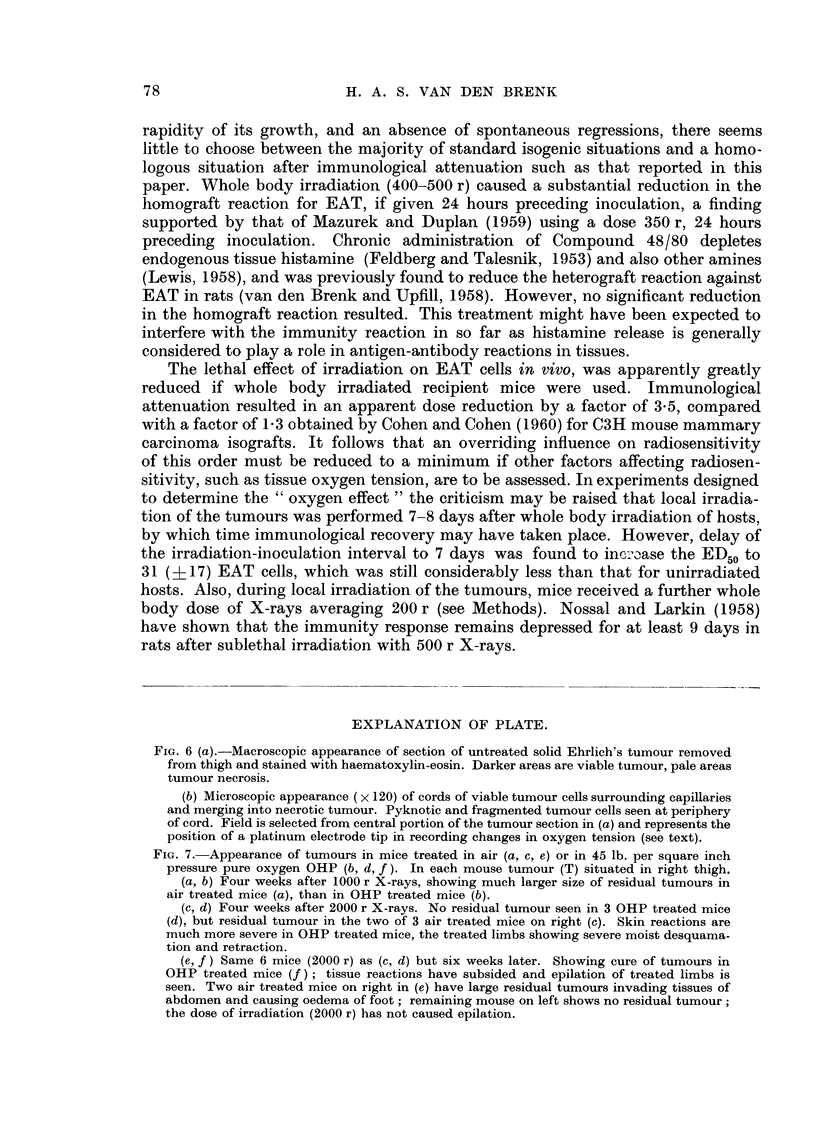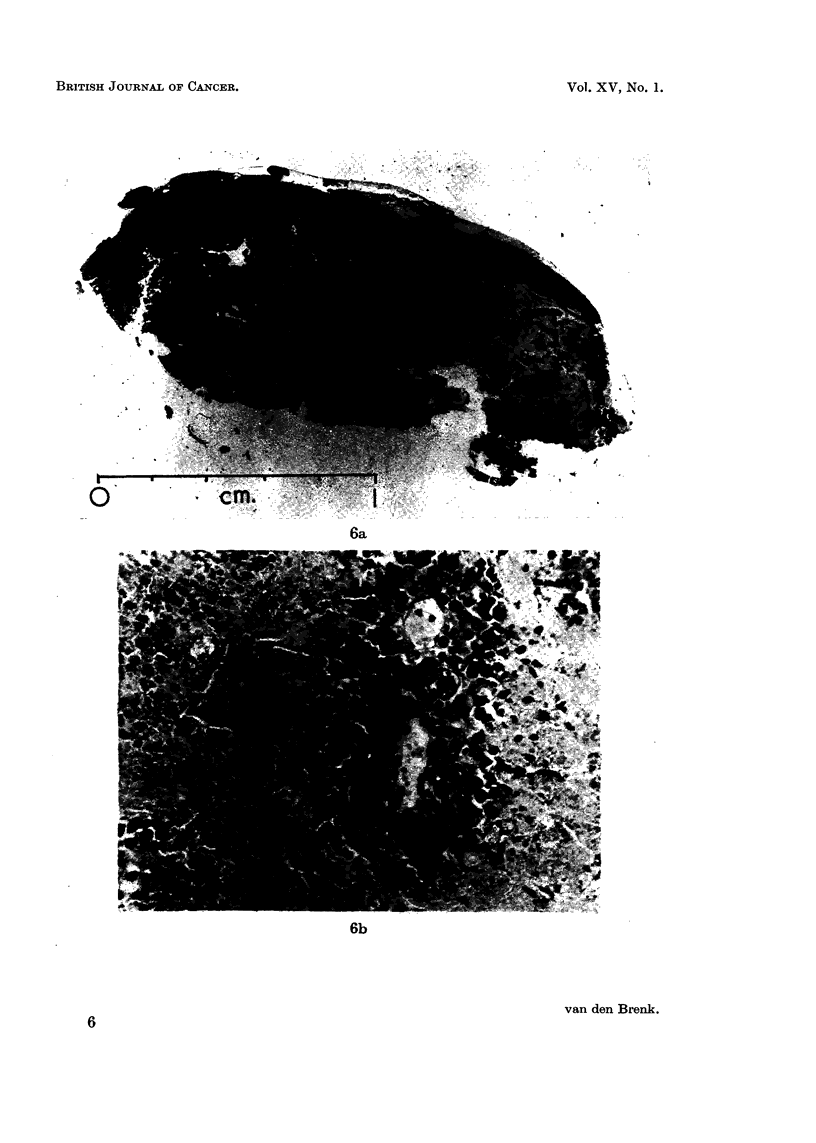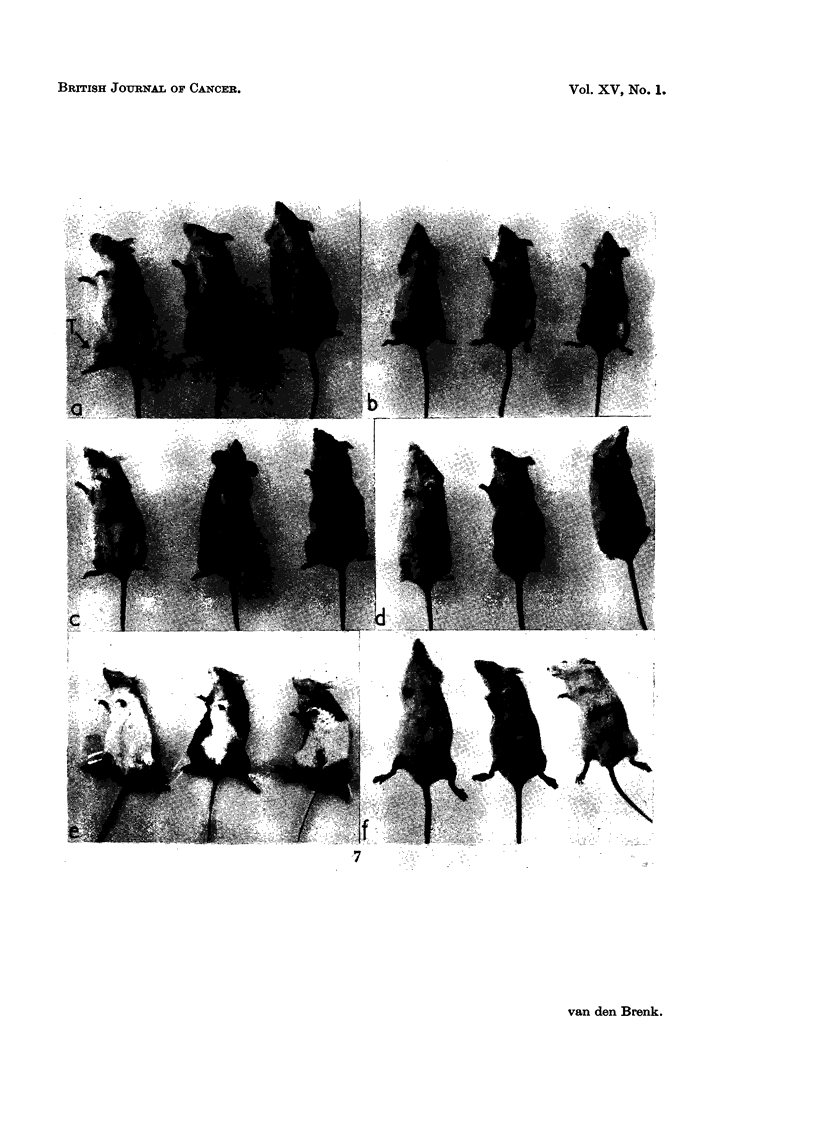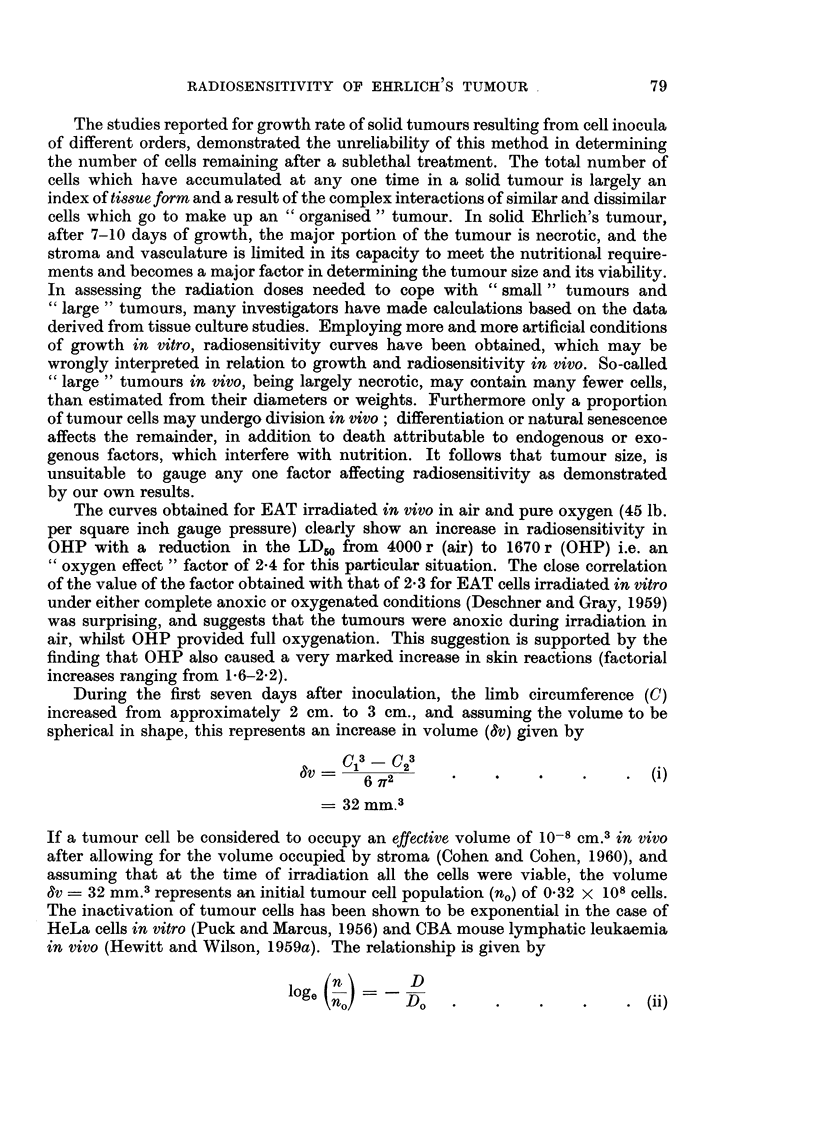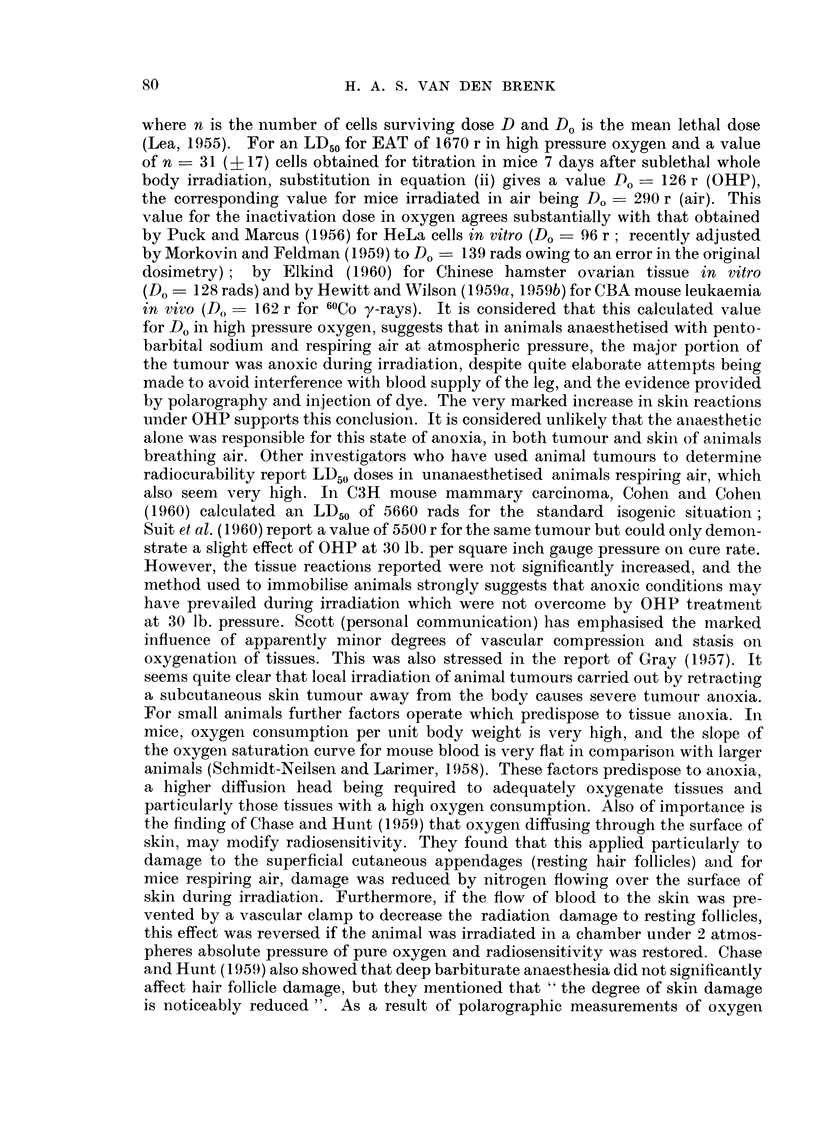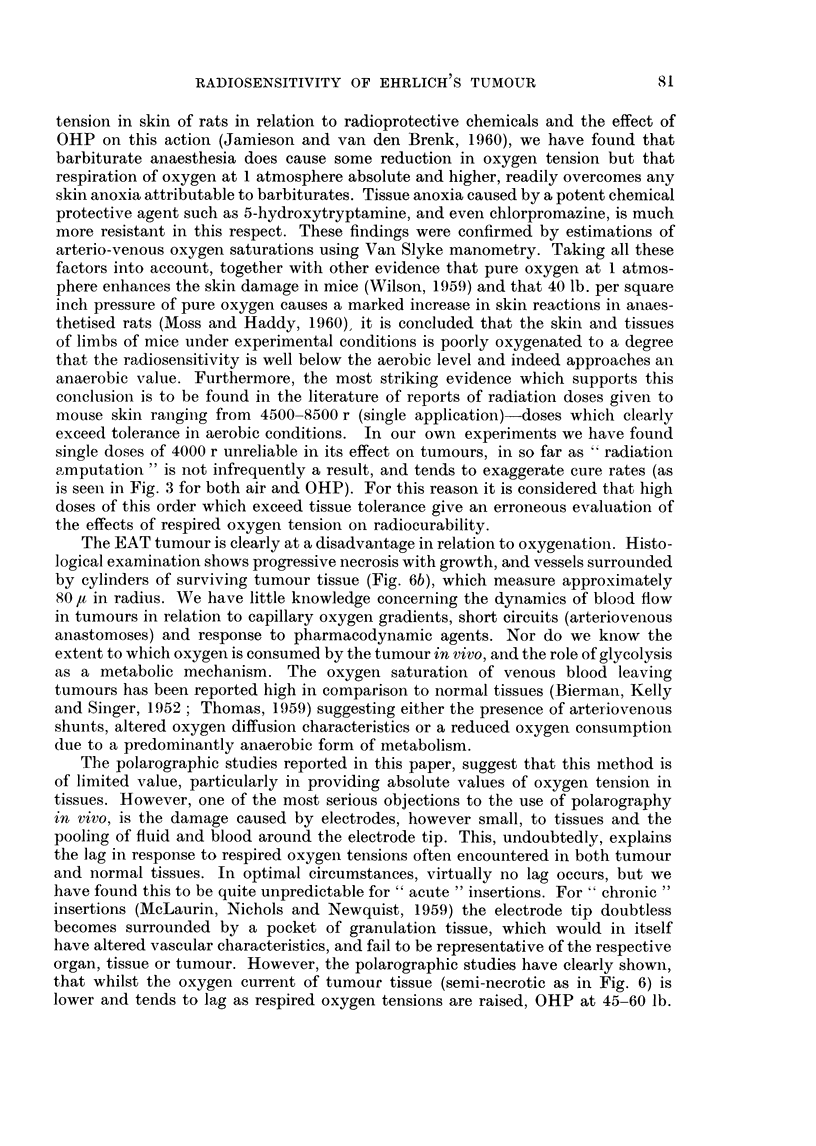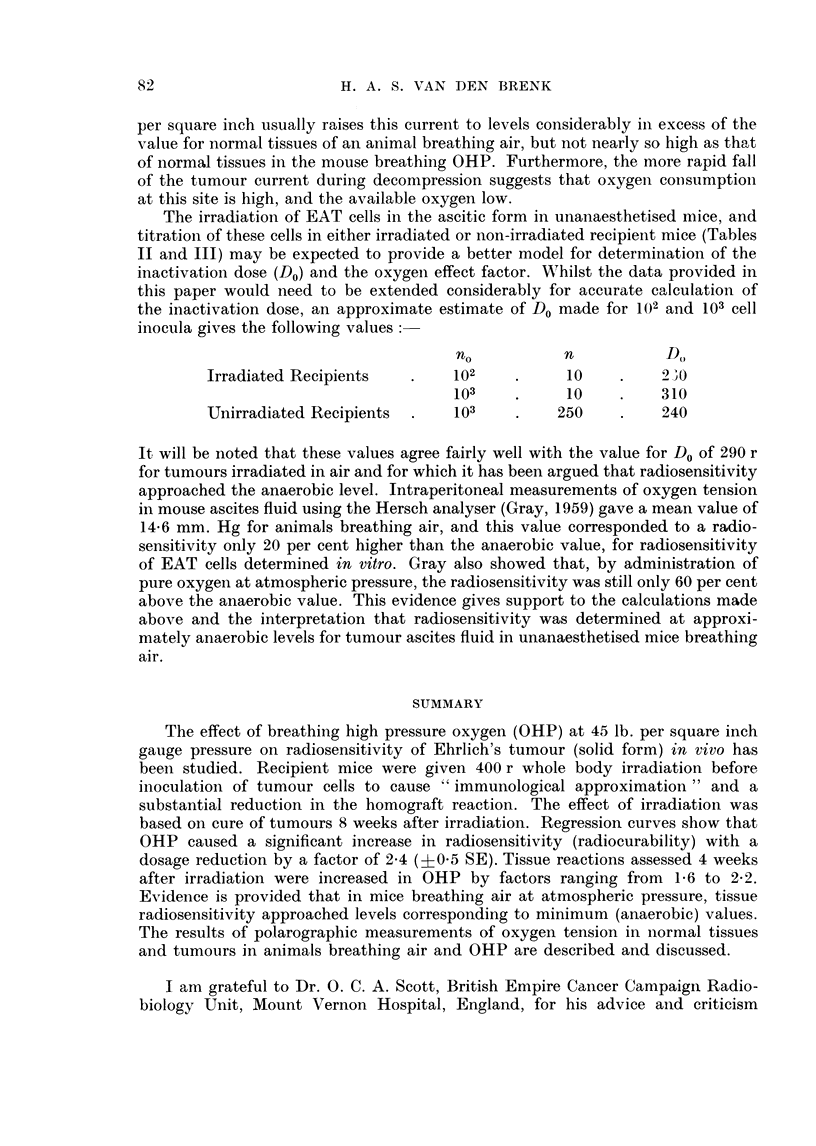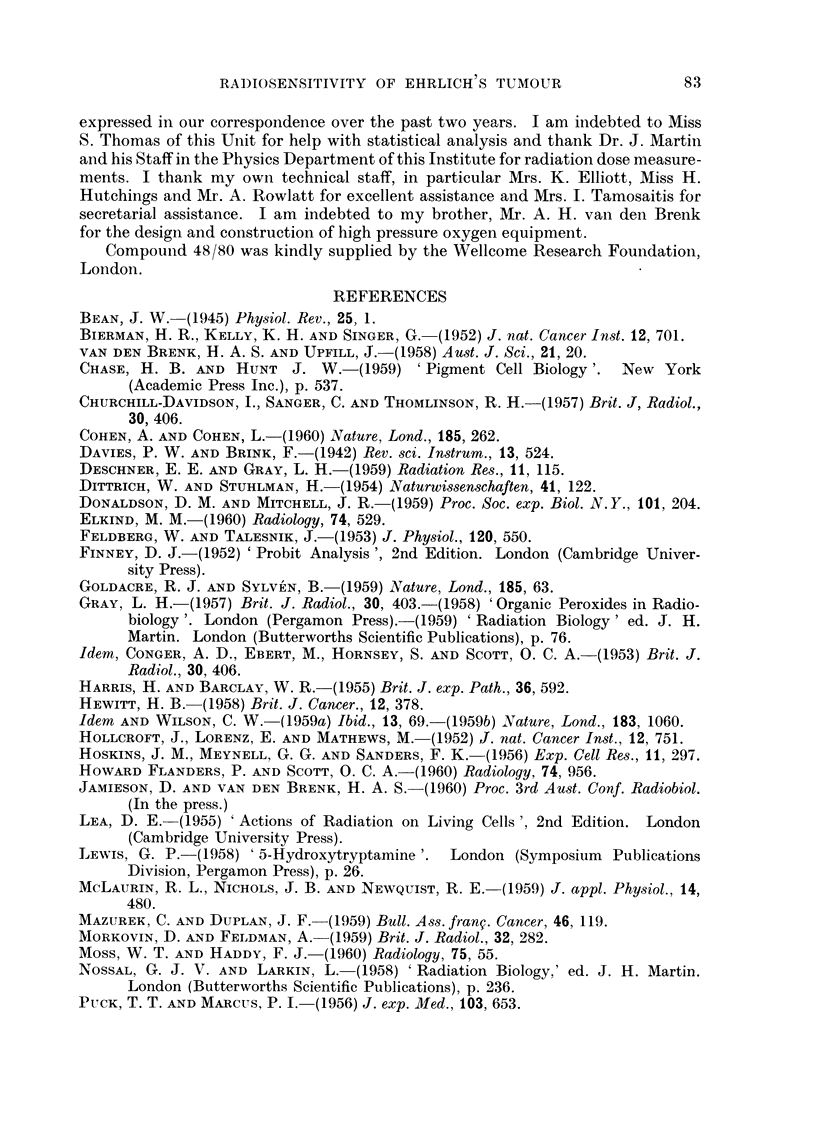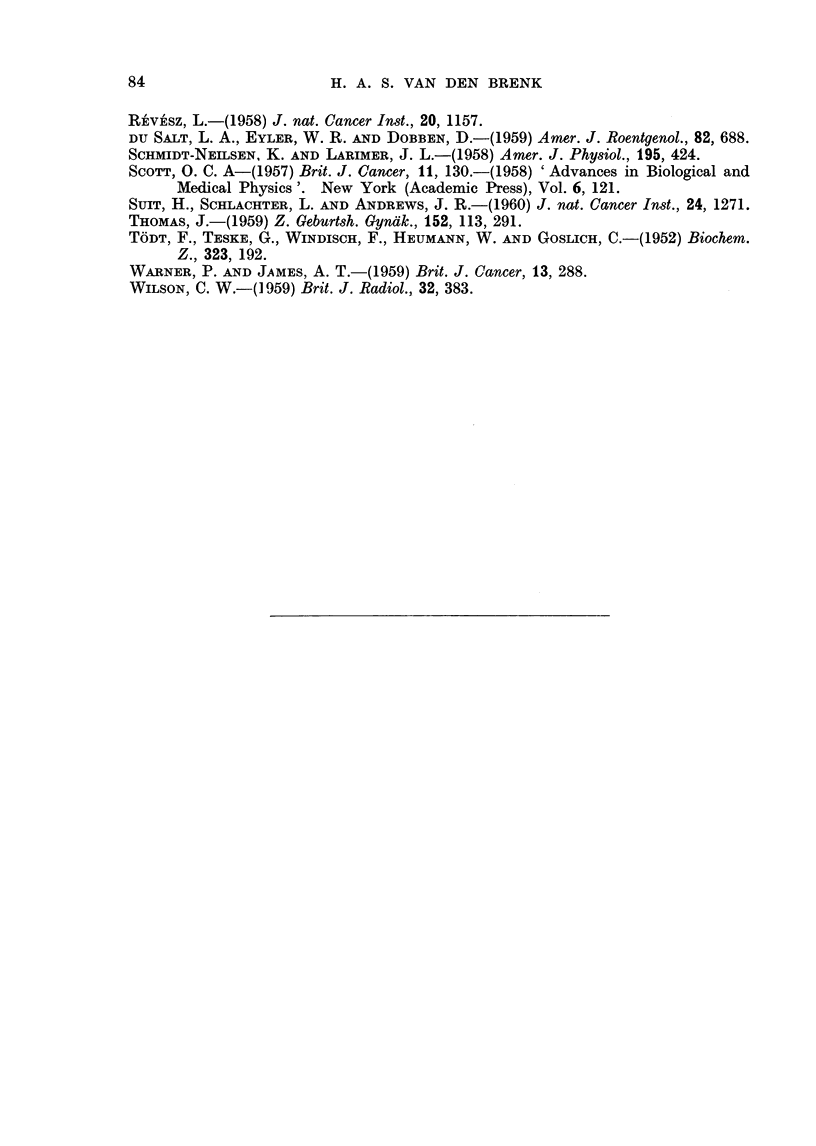# Effect of High Pressure Oxygen on Radiosensitivity of Ehrlich's Tumour in Mice After “Immunological Approximation”

**DOI:** 10.1038/bjc.1961.9

**Published:** 1961-03

**Authors:** H. A. S. van den Brenk

## Abstract

**Images:**


### EFFECT OF HIGH PRESSURE OXYGEN ON RADIOSENSITIVITY OF EHRLICH'S TUMOUR IN MICE AFTER " IMMUNOLOGICAL APPROXIMATION "

H. A. S. VAN DEN BRENK
*Radiobiological Research Unit, Cancer Institute Board, Melbourne, Australia*

Received for publication January 4, 1961

THE radiosensitivity of both normal and neoplastic cells is increased if oxygen is present during irradiation (see Gray, 1958, for summary). On the assumption that tumours *in vivo* contain cells in a state of anoxia relative to normal tissues when air at atmospheric pressure is being respired by the host, breathing of pure oxygen at raised pressures during irradiation has been employed with a view to increasing the therapeutic ratio (Hollcroft, Lorenz and Mathews, 1952 ; Gray *et a.*, 1953 ; Dittrich and Stuhlman, 1954 ; Churchill-Davidson, Sanger and Thomlinson, 1957 ; du Sault, Eyler and Dobben, 1959). However, the validity of this hypothesis is difficult to establish experimentally and the problem has been discussed in recent reviews (Scott, 1958 ; Howard Flanders and Scott, 1960). Difficulties are encountered in immobilising conscious animals without causing compression of the tumour blood supply during irradiation. The need to employ anaesthetics and their side effects, adds further problems, as does the difficulty in providing adequate radiation dosimetry of such tumours. However, certain biological factors are of even greater significance. In using transplantable tumours, the homograft reaction complicates the situation by making radiocurability less difficult in certain circumstances. Cohen and Cohen (1960) have shown that even for an apparently isogenic situation concerning the C3H mouse mammary adenocarcinoma, previous immunological attenuation of the host by either whole body irradiation or corticosteroids, increases the $LD_{50}$ curative X-ray dose from 5660 to 7500 rads and the estimated critical cell number (ED50) is reduced from 25 cells to approximately 1 cell. This suggests that in these experiments tumour and host were not, in fact, strictly isogenic, and some homograft reaction was operative.

Since the use of a readily available transplantable tumour, such as Ehrlich's ascites tumour has many technical advantages, experiments are reported in which this tumour was used as a homograft in hybrid mice after immunological approximation of the hosts, to determine the effect of high pressure oxygen respired by the treated animals, on tumour radiosensitivity and therapeutic ratio. This report includes initial experiments performed to determine the effect of whole body irradiation of inoculated mice on the " take " of Ehrlich's tumour.

### MATERIALS AND METHODS

### *Animals and ascites tumour*

Adult Walter and Eliza Hall hybrid mice weighing 40 g. were used for tumour inoculation. The mice were housed, six animals per cage, in an air conditioned animal house at 21 $\pm$ 1° C. Different coloured paints were used as skin marks to distinguish groups of mice and individual mice, to allow cross caging, and scoring of tumour incidence, growth rates and tissue reactions.

The tumour was Ehrlich's ascites tumour (hyperdiploid line ELD Lettrè, 46 chromosome mode), henceforth called EAT, previously passaged at weekly intervals in C3H mice for over 3 years in these laboratories.

*Inoculation*

All inoculations were made in the hind limbs of mice, just proximal to the knee joint. For each inoculation a volume of 0·2 ml. of tumour cell suspension was slowly injected into the thigh muscle. To prepare the suspension, a seven day ascites growth of the tumour was harvested from donor mice. The cells for each sample were counted in a haemocytometer chamber, and a viability test performed using the eosin exclusion technique of Shrek according to the method of Hoskins, Meynell and Sanders (1956); samples in which more than 5 per cent of cells stained pink were discarded. Appropriate dilutions of EAT cells for inoculation were made in ice cold Tyrode solution, to give cell concentrations ranging from $10^1$–$10^6$ EAT cells per 0·2 ml. inoculum.

*Preparation of recipient mice prior to inoculation*

One of three methods of preparing recipient mice were used :—

(*a*) No treatment (immunologically competent group)

(*b*) Chronic depletion of endogenous amines with Compound 48/80, given for 9 days prior to EAT inoculation. Increasing doses were administered twice daily in accordance with footnote to Table I.

(*c*) Whole body X-irradiation in doses of 400 r or 500 r given 24 hours preceding inoculation with EAT cells. The radiation was delivered by a constant potential X-ray source operated at 250 kv, 15 mA, 30 cm. FSD, and 1 mm. Cu HVL giving a dose rate in air of 300 r per minute.

*Irradiation of tumour cells* in vivo.

Groups of untreated and whole body irradiated recipient mice were inoculated with donor cells, previously exposed to graded doses of irradiation *in vivo*. The latter was accomplished by taking 10 donor mice with a ten day ascitic growth of EAT, and subjecting the animals to whole body irradiation. Viability counts (eosin exclusion index) were performed to check donor fluids before irradiation, and the unirradiated samples were pooled and titrated in groups of mice. The irradiation factors used to deliver whole body irradiation to these ascitic donor mice were 250 kv, 15 mA, 30 cm. FSD, 1 mm. Cu HVL giving a dose rate in air of 300 r per minute. Before irradiation the ten mice were placed in the same container which was surrounded with bolus to give full back scatter. The radiation exposure was interrupted after 200, 400, 800, 1600 and 2400 r had been administered, so that 2 mice could be removed and samples of tumour fluid withdrawn, counted, pooled and diluted for titration in groups of recipient mice. At each interruption of the exposure, the two mice selected at random were removed, marked, ascitic fluid withdrawn, the animals replaced and the exposure continued. A pair of different animals was used for each successive removal of irradiated cells.

TABLE I.—*Effect of Sub-lethal Whole Body X-irradiation (400 r) and Histamine Depletion (Compound 48/80 for 8 Days)* on Growth Rate and "Take" of EAT Cells in Female Hybrid Mice*

| Inoculum size EAT cells | Group | Mean increase in circumference of leg (cm.) (Days after inoculation) 7 | 14 | 21 | 28 | Fraction recipients with tumours 8 weeks after inoculation† |
|---|---|---|---|---|---|---|
| $10^6$ | Unirradiated | 1·4 | 2·4 | 3·3 | 4·4 | 6/6 |
| | Irradiated | 1·1 | 2·4 | 3·5 | 4·8 | 6/6 |
| | 48/80 | 1·5 | 2·7 | 3·3 | 4·3 | 6/6 |
| $10^5$ | Unirradiated | 0·8 | 2·0 | 3·0 | 4·4 | 6/6 |
| | Irradiated | 0·8 | 2·2 | 3·2 | 4·3 | 6/6 |
| | 48/80 | 0·7 | 2·2 | 3·3 | 3·8 | 6/6 |
| $10^4$ | Unirradiated | 0·3 | 0·7 | 0·8 | 1·3 | 5/6 |
| | Irradiated | 0·3 | 0·9 | 1·5 | 2·2 | 6/6 |
| | 48/80 | 0·4 | 0·6 | 1·2 | 1·6 | 4/5 |
| $10^3$ | Unirradiated | 0·2 | 0·3 | 0·4 | 0·9 | 4/6 |
| | Irradiated | 0·2 | 0·5 | 1·0 | 1·2 | 5/6 |
| | 48/80 | 0·1 | 0·3 | 0·4 | 0·6 | 2/6 |
| $10^2$ | Unirradiated | 0·2 | 0·3 | 0·1 | 0·3 | 1/6 |
| | Irradiated | 0·2 | 0·3 | 0·7 | 1·1 | 5/6 |
| | 48/80 | 0·1 | 0·3 | 0·2 | 0·3 | 1/6 |

* Dose schedule compound 48/80 (intraperitoneal injections) was as follows : Day (1) 1 × 30 μg. ; (2) 2 × 40 μg. ; (3) 2 × 50 μg. ; (4) 2 × 60 μg. ; (5) 2 = 70 μg. ; (6), (7) and (8) 1 × 80 μg. ; and inoculations on day 9.

† Mice developing large tumours during the observation span were sacrificed after 4 weeks but scored as positive.

*Local irradiation of tumours—oxygen pressurisation*

In these experiments each inoculation consisted of $10^6$ EAT cells injected into the right thigh of the mouse. The tumour was allowed to grow for 7–8 days, by which time the limb circumference had increased by approximately 10 mm. and contained a hard palpable tumour which distended the thigh as a spindle-shaped swelling. Calculations based on this average increase in size of limbs gives a tumour volume of approximately 1 c.c. at the time of irradiation. In over 1000 consecutive mice inoculated with $10^5$ or more EAT cells a palpable tumour was present in each animal 7–8 days after inoculation and no spontaneous regressions have been observed to date, whether recipient mice were untreated or "immunologically attenuated" as the result of whole body irradiation. A few mice in which tumours were considered undersize or oversize at 7–8 days, were rejected, as were mice in which the tumour had extended beyond the thigh into the buttock, so that its inclusion in the radiation field could not be ensured.

Mice were anaesthetised with sodium pentobarbital (70 mg. per kg. injected intraperitoneally) and irradiated in a steel pressure vessel of 35 litres capacity. The X-ray beam passed through a circular perspex portal 2·5 cm. thick, occupying the central portion of the upper lid of the chamber, which was retained in position by clamps when the chamber was pressurised. After decompression the clamps could be loosened and the lid elevated on guides and locked in position to introduce or remove animals from the chamber. To the undersurface of the lid an elevation platform was attached on which animal containers could be placed and raised to proximity with the undersurface of the perspex portal. The mouse tumours were irradiated in a special perspex container (see cross-section shown in Fig. 1). The animal body was placed in the outer well (a), and shielded with lead. This well contained 4 animals at each irradiation. The tumour bearing limb protruded through a semi-circular aperture in the lead shielding, into the central well, and was gently extended and fixed by a silk thread suture to a central pillar. The thread suture transfixed a 3 mm. wide strip of elastoplast, wrapped around the foot with minimal compression. The central well containing the 4 limbs was filled with bolus and the whole container placed beneath the perspex portal of the pressure vessel as shown. The top of the chamber was lowered, clamped in the closed position and the tumours irradiated through the perspex portal, whilst the chambeı contained either air at atmospheric pressure or pure oxygen under pressure. The radiation apparatus used generated X-rays at 250 kv, 15 mA, 30 cm. FSD, 1 mm. Cu HVL giving a dose rate in air of 300 r per minute. The mean tumour dose (TD) was calculated at point $y$ (Fig. 1) and calculations checked by direct measurements with ionisation chambers placed at points $a$, $x$, $y$ and $z$ as shown. The calculated and measured values were in close agreement. The tumour doses used varied from 500 r to 4000 r and were given as single exposures. The variation in dosage with depth in the tumour was less than $\pm 6$ per cent. However, animals received a whole body dose contribution (WBD), measured at point $a$, approximately 8·5 per cent of the mean TD, giving the following relationship between TD and WBD :—

| | | | | | |
|---|---|---|---|---|---|
| *TD* (*r*) | 500 | 1000 | 2000 | 3000 | 4000 |
| *WBD* (*r*) | 42 | 85 | 170 | 255 | 340 |

This whole body contribution resulting from irradiation of tumours must be added to the whole body dose (400 r) received 8 days previous (24 hours before inoculation).

During irradiation of tumours in animals breathing air at atmospheric pressure, a slow flow of air through the chamber was maintained. For irradiation of animals in oxygen under pressure, the chamber was flushed out with pure oxygen until the percentage oxygen concentration, measured on samples of exhaust gases with a Beckman Model D2 oxygen analyser, exceeded 95 per cent. Compression followed at a rate of 15 lb. per square inch per minute, till a preset level of 45 lb. per square inch gauge pressure (4 atmospheres absolute) was reached. This pressure was maintained in the apparatus during irradiation. Decompression, also at 15 lb. per square inch per minute, followed immediately after completing the radiation exposure. The ambient temperature in chamber changed by less than 1·5° C. during compression and decompression. Provision was not made for absorption of $CO_2$ within the chamber and was considered unnecessary owing to the negligible amount which accumulated. The chamber was provided with a series of electrically insulated contacts to allow polarographic measurements of oxygen tension to be made in tissues of animal under pressure (*vide infra*).

*Polarographic measurements of oxygen tension in tumours*

Since the tension of molecular oxygen which exists in the tumour is of paramount importance to the rationale of respiring oxygen at increased pressures as an adjunct to radiotherapy of tumours, polarographic measurements were made of oxygen tension in tumours and normal tissues of animals respiring oxygen at different pressures. Open type cathodes were made from platinum wire, 32 s.w.g., 0·23 mm. in thickness, and covered with Araldite insulation except for the terminal 1 mm. tip. These were inserted to various depths in the tumour, or in surrounding normal tissues. The circuit included a galvanometer, of variable sensitivity, used to measure the cathode current. The latter is considered to represent the oxygen tension adjacent to the exposed platinum surface (Davies and Brink, 1942). The circuit was completed using an anode consisting of Pb/standard acetate solution/KCl-Agar bridge (Tödt *et al.*, 1952), inserted in the rectum or subcutaneous tissues of the animal. Previous experiments to calibrate this system showed a plateau in the current-voltage calibration curve similar to that for the calomel half-cell anode (Davies and Brink, 1942). For the latter the mid-region of the plateau corresponded to an impressed potential of —0·60 volt, but for the Tödt anode this region corresponds to zero potential. The electrodes were calibrated in Hank's solution containing 20 per cent v/v sheep serum, equilibrated with various tensions of oxygen. The form of the oxygen-current curves obtained agreed with those of Harris and Barclay (1955) for $O_2$ tensions below 760 mm. Hg, with a linear relationship of the logarithm of the $O_2$ tension ($T$) to the current ($I$), such that

$$\log T = K_1 I + K_2$$

where $K_1$, $K_2$ are constants determined by the characteristics of the Pt electrode used. For $O_2$ tensions in excess of 760 mm. Hg, determined inside the pressure chamber at an ambient temperature of 19°C the relationship between $T$ and $I$ was essentially linear. However, conditions for calibration under pressure, could not be readily controlled in respect to temperature and water vapour pressure. Furthermore the time allowed (10 minutes) for equilibration of the calibration fluid with the gas phase may have been too short although the current had reached a maximum after 5 minutes using 2 ml. of fluid to give a depth of 3 mm. in a dish 3 cm. in diameter. The pressure vessel was rocked to give quite vigorous agitation of the fluid between measurements, taken at 2 minute intervals. Currents obtained for electrodes in tissues, showed great variability, according to the site and positioning of electrodes and their movements in tissue. Calibrated electrodes gave tissue oxygen tensions considerably in excess of values determined by other means for tissue fluids. However, the response of electrodes to changes in respired oxygen tension was consistent, being unaffected by moderate elevations in respired $CO_2$ and certain treatments not considered to interfere with tissue oxygen tensions. The relative changes in the current registered by electrodes placed in the tumour and normal tissues of anaesthetised mice respiring different tensions of $O_2$ were considered of sufficient significance to warrant the inclusion of representative records under results, together with additional details of the experimental technique.

*Tests for vascular compression*

Since any reduction in blood flow in the limb during irradiation resulting from positioning of the animal, would reduce the effects of an increase in respired oxygen tension, special care was taken to avoid stretching and compression of the limbs during treatment. At no stage was pallor or cyanosis noted in the skin of extended limbs. With the legs of anaesthetised mice held in the irradiation position (Fig. 1), an electrode was inserted in the popliteal region of each extended leg. The oxygen current was registered with animals successively breathing air, pure $O_2$, 5 per cent $O_2$ and air and faithful changes in current resulted. A further test was performed with anaesthetised mice in the irradiation position. Two ml. of lissamine green (Gurr No. 3591) solution was injected intraperitoneally, and the time of appearance of the green discoloration of the skin of extended and unextended limbs measured. No significant difference was observed for the two limbs nor were the results in unanaesthetised mice significantly different from those in anaesthetised animals.

FIG. 1.—Cross section through irradiation portal of pressure chamber and container for irradiating mouse legs (tumours). Percentage depth doses measured at points $a$, $x$, $y$ and $z$ with shielding and bolus in position are shown. Outer well of container normally holds four mice at a time during irradiation exposure, but only one is shown.

### *Tumour regression*

Results of treatment were assessed either as *cure* (no palpable tumour) 8 weeks after irradiation or as *growth rate* (circumference of limb) during 4 weeks after irradation. Limbs of survivors were palpated weekly after irradation, and presence or absence of palpable tumour charted. Mice dying within 14 days of irradiation of tumours, were excluded from the analysis, after which time a progressive record of the fraction of animals with tumours was made weekly. Mice surviving at 6 weeks with tumours 40 mm. or more in circumference were killed and scored as failed cures at 8 weeks. Mice in which the irradiated tumour disappeared, but extension appeared proximal to the irradiated zone were scored as failed cures.

The maximum limb circumference at the site of the tumour was measured by means of a silk loop protruding from the end of a transfusion needle and held taut by a loose trocar fitted inside the canula. The loop was withdrawn from the limb and the doubled length of silk measured against a rule. This method has proved much more reproducible than the use of calipers in measuring size of limb tumours.

### *Tissue reactions*

A time 4 weeks after irradiation was chosen to assess tissue reactions in irradiated limbs, which were scored and given an arbitrary numerical index of severity as follows :—

| | |
|---|---|
| Partial epilation . . . . . . . | 1 |
| Complete epilation . . . . . . . | 2 |
| Superficial skin loss . . . . . . | 3 |
| Skin loss and oedema . . . . . . | 4 |
| Partial sloughing (below knee) . . . . | 5 |
| Partial sloughing (below and above knee) . . | 6 |
| Complete sloughing (" radiation amputation ") . | 7 |

Tissue reactions in mice irradiated in oxygen under pressure (OHP) and air corresponding to each dose, were compared by calculating the mean " severity index ", $X_{\text{OHP}}$ and $X_{\text{air}}$ respectively, and using the ratio,

$$t = \frac{X_{\text{OHP}}}{X_{\text{air}}}$$

where $t$ is the " oxygen effect factor " for each dose level.

*Statistical analysis*

Probit analysis (Finney, 1952) was used to calculate regression equations, and to determine the significance of observed differences in cure rate in animals breathing air, and oxygen under pressure. Other statistical procedures such as the calculation of variances, students $t$ values and the $\chi^2$ test, were carried out in accordance with standard practice.

### RESULTS

*Inoculation of irradiated and unirradiated hosts*

(a) *Unirradiated cells.*—Limbs of mice were inoculated with graded numbers of EAT cells in $\log_{10}$ dilutions from $10^6$ to 10 cells, and the tumours scored which developed during a period of 50 days after inoculation. The number of cells required to produce 50 per cent of tumours ($ED_{50/50}$ days) was calculated from probit regression curves. In untreated mice the $ED_{50/50}$ was 281 ($\pm$71 SE) cells ; in mice irradiated with 500 r whole body X-ray dose 24 hours before inoculation the $ED_{50/50}$ was reduced to $<$10 cells. Increase of the irradiation-inoculation interval to 7 days increased the $ED_{50/50}$ to 31 ($\pm$18) cells.

To determine the effect of " immunological attenuation " by whole body irradiation on the growth rate of EAT in legs of mice, tumour cells were titrated in the limbs of (i) unirradiated mice ; (ii) mice receiving 400 r, 24 hours preceding inoculation, and (iii) mice treated with increasing doses of compound 48/80 for 8 days preceding inoculation, to deplete tissue histamine (Feldberg and Talesnik, 1953). This last group was included for two reasons ; firstly to act as a further control group, and secondly in view of a previous finding that in chronic histamine depleted rats a heterologous graft of Ehrlich's tumour grew more rapidly, as a temporary graft after inoculation (van den Brenk and Upfill, 1958). The limb sizes in the various groups were measured weekly for 4 weeks after inoculation. Also the total incidence of tumours which had developed in 8 weeks was recorded. The results are given in Table I, Fig. 2 and show that for larger inocula ($10^5$, $10^6$ cells) the mean rate of tumour growth (and tumour incidence) is affected neither by whole body irradiation nor by histamine depletion. However, for smaller inocula ($<10^4$ cells), the mean rate of growth was clearly increased by whole body irradiation, but not by histamine depletion, and was paralleled by an increase in tumour incidence. However, the growth curves show how difficult it is to relate quantitatively, growth rate of a tumour *in vivo*, to the number of viable cells initiating such growth. Furthermore, the standard deviations for measurements of tumours resulting from small inocula, were much greater than for larger inocula, owing to tumours not developing in some animals, whilst in remaining animals tumours often grew almost as rapidly as those in animals which had received much larger inocula.

FIG. 2.—Growth curves for Ehrlich's tumour in mouse limbs, following titration of cells in untreated mice, mice previously treated with Compound 48/80 (" subacute depletion ", see text) and mice which received 400 r whole body X-irradiation 24 hours before inoculation.

(b) *Irradiated cells*.—Parallel numbers of EAT cells were irradiated with doses of X-rays *in vivo*, and inoculated into the limbs of either unirradiated mice or mice which had received 500 r whole body irradiation, 24 hours preceding inoculation. The results are set out in Table II, and show a marked increase in survival of EAT cells in irradiated recipient mice. Calculation of the doses ($LD_{50}$) received by EAT cells *in vivo* to give 50 per cent incidence of tumours in mice for respective sizes of inoculum are set out in Table III. The $LD_{50}$ dose was approximately 3·5 times higher if irradiated (immunologically attenuated) recipient mice were used. In other words, the radiation dose necessary to sterilise EAT cells *in vivo* may be underestimated by a factor of 3·5 in the homologous situation used in this experiment, if the recipient mice are untreated, in comparison to " immunologically attenuated " mice.

TABLE II.—*Titration of Ehrlich Ascites Tumour Cells Irradiated* In Vivo *in ascitic Form, in Mice Subjected to* (a) *no Previous Irradiation, and* (b) 500 *r Whole Body Irradiation* 24 *hours Preceding Inoculation. The Cells were Inoculated into Legs of Recipient Mice, and Tumour Incidence Scored at* 53 *Days**

| Number of cells inoculated | Irradiation dose *in vivo* | Tumour incidence—fraction (%) Untreated recipients | Irradiated recipients |
|---|---|---|---|
| $10^2$ | 0 r | 5/12 (42) | 9/9 (100) |
| | 200 r | 4/8 (50) | 8/8 (100) |
| | 400 r | 1/8 (12) | 4/6 (67) |
| | 800 r | 2/8 (25) | 3/5 (60) |
| | 1600 r | 0/8 (0) | 1/8 (12) |
| | 2400 r | 0/8 (0) | 0/8 (0) |
| $10^3$ | 0 r | 9/10 (90) | 8/8 (100) |
| | 200 r | 3/5 (60) | 8/8 (100) |
| | 400 r | 4/8 (50) | 7/7 (100) |
| | 800 r | 3/8 (37) | 4/7 (57) |
| | 1600 r | 1/8 (12) | 5/8 (62) |
| | 2400 r | 0/8 (0) | 1/8 (12) |
| $10^4$ | 0 r | 20/20 (100) | 12/12 (100) |
| | 200 r | 6/7 (86) | 8/8 (100) |
| | 400 r | 8/8 (100) | 7/7 (100) |
| | 800 r | 8/8 (100) | 3/3 (100) |
| | 1600 r | 2/8 (25) | 7/7 (100) |
| | 2400 r | 0/8 (0) | 6/8 (75) |
| $10^5$ | 0 r | 28/28 (100) | 10/10 (100) |
| | 200 r | 8/8 (100) | 7/7 (100) |
| | 400 r | 8/8 (100) | 8/8 (100) |
| | 800 r | 8/8 (100) | 6/6 (100) |
| | 1600 r | 6/8 (75) | 8/8 (100) |
| | 2400 r | 7/8 (87) | 7/7 (100) |

* Recipient mortality over 53 days after inoculation 8/234 = 2·6 per cent for unirradiated mice, 35/216 = 16 per cent for irradiated mice.

TABLE III.—*$LD_{50}$ Doses for* $10^2$–$10^5$ *Cell Inocula in Non-Irradiated and Irradiated* (500 *r*) *Recipient Mice Respectively* (*from Data Table II*). *Dosage Reduction Factor* R *calculated from*

$$R = \frac{LD_{50}}{LD_{50}}\left(\frac{\text{irradiated recipients}}{\text{non-irradiated recipients}}\right)$$

| Inoculum size of EAT cells | Recipients | $LD_{50}$ | *R* |
|---|---|---|---|
| $10^2$ | Non-irradiated | 200 r | 3·6 |
| | Irradiated | 720 r | 3·6 |
| $10^3$ | Non-irradiated | 340 r | 3·5 |
| | Irradiated | 1200 r | 3·5 |
| $10^4$ | Non-irradiated | 1050 r | — |
| | Irradiated | (insufficient data) | — |
| $10^5$ | Non-irradiated | (insufficient data) | — |
| | Irradiated | (insufficient data) | — |

*Effect of high pressure oxygen (OHP) on radiosensitivity*

(a) *Curative results.*—Previous experiments using untreated host mice, gave very variable dose-effect curves for the cure of solid Ehrlich's tumour by X-radiation, and in the present experiment, all mice received 400 r whole body X-radiation 24 hours preceding inoculation of limbs with $10^6$ EAT cells. The apparent cure rate of tumours at various intervals after local irradiation of 7–8 day old tumours in anaesthetized mice breathing either air at atmospheric pressure or pure oxygen at 45 lb. per square inch gauge pressure (4 atmospheres absolute) is given in Table IV. The overall cure rate (52 per cent) estimated at 8 weeks after irradiation for mice breathing OHP was significantly better ($p < 0{\cdot}001$) than that (18 per cent) for animals breathing air.

TABLE IV.—*Resolution of Palpable Tumours (Cure Rate) in Hind Limbs of Inoculated Mice Following X-irradiation. All Animals Received 400 r Whole Body Irradiation 24 Hours Before Inoculation with $10^6$ Ehrlich Ascites Tumour Cells, and the Growing Tumours were Irradiated under Pentobarbital Anaesthesia 7 or 8 Days Later with Mice Breathing Either Air at Atmospheric Pressure, or Pure Oxygen at 45 lb. per Square Inch Gauge Pressure (OHP)*

| Tumour dose and gas respired | Fraction cured (%) at stated times after irradiation: 14 days | 28 days | 6 weeks | 8 weeks | Fraction of mice (%) dead from 2 to 6 weeks after irradiation |
|---|---|---|---|---|---|
| 500 r in air | 0/5 (0) | 0/5 (0) | 0/3 (0) | 0/3 (0) | 2/5 (40) |
| 500 r in OHP | 0/10 (0) | 0/9 (0) | 0/8 (0) | 0/8 (0) | 2/10 (20) |
| 1000 r in air | 0/39 (0) | 1/20 (5) | 1/17 (6) | 1/15 (7) | 22/39 (56) |
| 1000 r in OHP | 3/42 (7) | 3/19 (16) | 3/11 (27) | 3/8 (37) | 31/42 (74) |
| 1500 r in air | 1/27 (6) | 1/19 (5) | 1/15 (7) | 1/15 (7) | 12/27 (44) |
| 1500 r in OHP | 13/28 (46) | 8/19 (42) | 6/12 (50) | 4/10 (40) | 16/28 (57) |
| 2000 r in air | 4/37 (11) | 3/33 (9) | 4/28 (14) | 3/24 (12) | 9/37 (24) |
| 2000 r in OHP | 28/37 (76) | 19/30 (63) | 13/23 (56) | 12/22 (55) | 14/37 (38) |
| 3000 r in air | 12/43 (51) | 16/35 (46) | 11/27 (41) | 6/24 (25) | 16/43 (37) |
| 3000 r in OHP | 16/26 (62) | 16/19 (84) | 10/12 (83) | 8/10 (80) | 14/26 (54) |
| 4000 r in air | 6/10 (60) | 8/9 (89) | 8/9 (89) | 5/6 (83) | 1/10 (10) |
| 4000 r in OHP | 6/10 (60) | 10/10 (100) | 7/7 (100) | 6/6 (100) | 3/10 (30) |
| All doses in air | 33/161 (20) | 29/121 (24) | 25/99 (25) | 16/87 (18)* | 62/161 (39)† |
| All doses in OHP | 66/153 (43) | 56/106 (53) | 39/73 (55) | 33/64 (52)* | 80/153 (52)† |

* Total lethalities, 8 weeks after irradiation were 41·8 per cent (71/170) for mice treated in air, and 55·7 per cent (92/165) for mice treated in OHP. The difference is probably significant ($\chi^2 = 3{\cdot}5$, $p = 0{\cdot}05$) as is that for total deaths occurring between second and sixth weeks† ($\chi^2 = 3{\cdot}4$, $p = 0{\cdot}05$).

† Total cure rate significantly higher in OHP ($\chi^2 = 10{\cdot}9$, $p < 0{\cdot}001$).

Calculation of probit regression equations for the log dose-effect relationship for the two groups, gave regressions which were essentially parallel and given by—

$$y = 3{\cdot}40x + 1{\cdot}94 \quad \text{(air)}$$

$$y = 3{\cdot}40x + 3{\cdot}22 \quad \text{(OHP)}$$

Comparison of the regressions, gives a dosage reduction factor of 2·4 (range of one standard error is 2·9, 1·9). The calculated regressions are plotted on a linear scale in Fig. 3.

In this experiment, 335 tumours were irradiated, 170 in air and 165 in OHP respectively. Six weeks after irradiation of tumours, only 99 animals survived in the groups treated in air, and 73 in OHP treated groups, giving lethalities of 41·8 and 55·7 per cent respectively 6 weeks after irradiation of tumours. The high lethality rate is variously attributed to whole body irradiation, severe local tissue

FIG. 3.—Calculated regression curves for percentage cure rate at 8 weeks after irradiation of Ehrlich's tumour in " immunologically attenuated " mice breathing either air (atmospheric pressure) or pure oxygen (at 45 lb. per square inch gauge pressure) during irradiation.

reactions in higher dosage groups, the toxic effects of spontaneous tumour necrosis and to a lesser extent metastases which were observed in approximately 2 per cent of the treated mice. Whilst the lethality was higher in the OHP treated group, despite the higher cure rate of tumours in surviving animals, this increase is not considered largely due to any toxic effects of oxygen (e.g. pulmonary damage). The high radiation dose rate used enabled exposure of animals to OHP to be kept to a minimum and barbiturate anaesthesia also provides marked protection against oxygen poisoning (Bean, 1945). In our animals convulsions under OHP did not occur and very few deaths were recorded within 72 hours of exposure to OHP. Local tissue reactions were more severe in this group (*vide infra*) and are considered a more important contribution to lethality than oxygen poisoning.

However, careful post mortem examinations to ascertain causes of death were not performed.

(b) *Effects on tumour growth rate.*—The size of tumours after irradiation, were noticeably smaller in the OHP treated groups. In Table V measurements made

TABLE V.—*Measurements of Circumferences of Normal (Left) Limbs and Right Limbs Inoculated with* $10^6$ *Cells of Ehrlich Ascites Tumour, Before and After X-irradiation of Tumours. Mice Breathing Either Air or Pressurised Oxygen, OHP (45 lb. per Square Inch Gauge Pressure) During Irradiation. All Animals Received* 400 *r Whole Body Irradiation* 24 *Hours Preceding Inoculation with Tumour Cells. Tumours Locally Irradiated* 7 *Days After Inoculation*

| Irradiation treatment (number of mice) | Side | Limb circumference ±SE in mm. at times (days) after inoculation: 7 | 14 | 21 | 28 | Surviving fraction mice with palpable tumours (28 days after inoculation) |
|---|---|---|---|---|---|---|
| Unirradiated (15) | R. | 34 ± 0·6 | 44 ± 1·0 | 52 ± 3·4 | 68 ± 4·7 | 14/14 |
| | L. | 24 ± 0·3 | .. | .. | .. | |
| 500 r in air (9) | R. | 35 ± 0·6 | 46 ± 0·9 | .. | >60 | 8/8 |
| | L. | 24 ± 0·6 | .. | .. | .. | |
| 500 r in OHP (8) | R. | 33 ± 0·7 | 43 ± 1·0 | .. | >60 | 8/8 |
| | L. | 25 ± 0·4 | .. | .. | .. | |
| 1000 r in air (10) | R. | 32 ± 0·6 | 39 ± 1·7 | 39 ± 1·5 | 42 ± 2·3 | 9/9 |
| | L. | 23 ± 0·4 | .. | .. | .. | |
| 1000 r in OHP (9) | R. | 33 ± 0·8 | 30 ± 0·9 | 31 ± 0·7 | 35 ± 3·4 | 5/7 |
| | L. | 24 ± 0·4 | .. | .. | .. | |
| 2000 r in air (10) | R. | 36 ± 1·0 | 33 ± 1·2 | 38 ± 1·6 | 42 ± 3·4 | 7/10 |
| | L. | 26 ± 0·5 | .. | .. | .. | |
| 2000 r in OHP (10) | R. | 35 ± 0·9 | 31 ± 0·9 | 31 ± 1·0 | 32 ± 1·0* | 3/9 |
| | L. | 25 ± 0·5 | .. | .. | .. | |
| 3000 r in air (10) | R. | 34 ± 0·6 | 32 ± 1·5 | .. | .. | 3/9 |
| | L. | 24 ± 0·4 | .. | .. | .. | |
| 3000 r in OHP (10) | R. | 33 ± 0·8 | 30 ± 1·0 | .. | .. | 0/8 |
| | L. | 25 ± 0·4 | .. | .. | .. | |
| 4000 r in air (10) | R. | 32 ± 0·6 | 30 ± 0·9 | .. | .. | 1/9 |
| | L. | 24 ± 0·3 | .. | .. | .. | |
| 4000 r in OHP (10) | R. | 34 ± 0·5 | 31 ± 0·6 | .. | .. | 0/7 |
| | L. | 24 ± 0·5 | .. | .. | .. | |

* Severe skin reactions in 3/9 mice, prevented measurement of limbs. In 3000 r and 4000 r groups severity of reactions also prevented measurements of 21 and 28 day tumour sizes.

weekly for 3 weeks after irradiation are recorded for tumour doses ranging from 500 to 4000 r, administered to mice in either air or OHP. It is seen that repeated measurements over this 3 week period, could only be made with any accuracy for doses less than 2000 r owing to local tissue reactions. In Fig. 4 growth curves are plotted for control (unirradiated) tumours and tumours irradiated with 1000 r and 2000 r in either air or OHP. It is seen that irradiation in $O_2$ is the more effective. Comparison of the tumour sizes, 14 and 21 days after irradiation in air or OHP, give the following levels of significance—

| | | | | |
|---|---|---|---|---|
| 14 days | 1000 r | $t = 4{\cdot}82$ | (df = 14) | $p < 0{\cdot}001$ |
| | 2000 r | $t = 3{\cdot}70$ | (df = 17) | $p = 0{\cdot}002$ |
| 21 days | 1000 r | $t = 1{\cdot}71$ | (df = 14) | ns |
| | 2000 r | $t = 2{\cdot}82$ | (df = 14) | $0{\cdot}02 > p > 0{\cdot}01$ |

FIG. 4.—Growth curves for control tumours and tumours irradiated with 1000 r or 2000 r in either air or oxygen (45 lb. per square inch gauge pressure). All mice received 400 r whole body X-irradiation 24 hours before inoculation. Vertical lines represent $\pm$ one standard error.

However, there is no significant difference in the size of tumours 14 and 21 days after irradiation in air, for 1000 r and 2000 r respectively, nor for these doses received by tumours in OHP. It is considered that tissue reactions complicated the situation and together with other considerations (*vide supra*), suggests that measurements of growth rate of solid tumours *in vivo* is quite unreliable as a quantitative index of radiation effects. Also only a narrow intermediate dose range can be utilised for measuring tumours in limbs of mice to determine modifications of radiosensitivity ; lower doses fail to reduce the cell population to sufficiently critical numbers and higher doses cause severe tissue reactions.

(c) *Effect of OHP on reaction of normal tissues.*—At an early stage in this investigation it became apparent that radiosensitivity of normal tissues was appreciably greater as the result of treating animals under high pressure oxygen. Subsequently an attempt was made to classify these reactions, and adopt an arbitrary scale of values to measure degrees of severity (see Methods). The average tissue reaction index $x$ ($\pm$SE) is given for tumour doses of 2000, 3000 and 4000 r administered in either air or OHP (Groups I, II and III, Table VI). An extra Group (Ia) was included in which recipient animals did not receive whole body irradiation prior to inoculation of EAT cells, but the tumours were irradiated with 2000 r either in air or in OHP.

TABLE VI.—*Tissue Reactions in Limbs Expressed as* x *units* ($\pm SE$) *Due to Irradiation of Tumours in Mice Breathing Either Air or Pressurised Oxygen* (45 *lb. Per Square Inch Gauge Pressure ; OHP*). *All Animals Except Those in Group Ia Received* 400 *r Whole Body Irradiation* 24 *Hours Preceding Inoculation with* $10^6$ *Ehrlich Ascites Tumour Cells*

| Group | Treatment (number of mice) | Tissue reaction ($x \pm$ SE) in arbitrary units | Radiosensitivity ratio ($t$)* |
|---|---|---|---|
| I | 2000 r in air (10) | 1.5 ± 0.2 | 2.0 |
| | 2000 r in OHP (9) | 3.0 ± 0.3 | |
| Ia | 2000 r in air (10) | 1·3 ± 0·2 | 2·2 |
| | 2000 r in OHP (10) | 2·9 ± 0·3 | |
| II | 3000 r in air (10) | 2·9 ± 0·4 | 1·6 |
| | 3000 r in OHP (8) | 4·6 ± 0·3 | |
| III | 4000 r in air (9) | 3·9 ± 0·4 | 1·6 |
| | 4000 r in OHP (10) | 6·3 ± 0·2 | |

* $t = \frac{X_{OHP}}{X_{air}}$. Mean value for Group I, II and III, $t = 1 \cdot 7$.

For all four groups, the tissue reaction ($x$) in OHP was significantly greater than that in air ($p < 0{\cdot}001$ for comparisons at each dose level). There was no significant difference between air treated animals for Groups I and Ia, nor between OHP treated animals in these groups, suggesting that "immunological attenuation" due to whole body irradiation failed to affect tissue reactions, and that the "oxygen effect" for normal tissues also was independent of the immunological situation for tumour and host. There were significant differences due to effect of dosage in both air treated and OHP treated groups; in each case $p < 0{\cdot}02$, with the exception of the comparison for 3000 r with 4000 r in air (Groups II and III) for which $p = 0{\cdot}1$.

Calculation of the tissue sensitivity factor ($t$) for air and OHP

$$\left(t = \frac{X_{OHP}}{X_{air}}\right),$$

for the four groups gave values ranging from 1·6 to 2·2.

The differences in tissue reactions ($S$) for OHP and air treated limbs at each dose level, gave the following values :—

| | $S = (X_{OHP} - X_{air})$ |
|---|---|
| Group I | $S = 1{\cdot}5 \pm 0{\cdot}36$ |
| Group Ia | $S = 1{\cdot}6 \pm 0{\cdot}36$ |
| Group II | $S = 1{\cdot}7 \pm 0{\cdot}50$ |
| Group III | $S = 2{\cdot}4 \pm 0{\cdot}45$ |

These values of $S$ for the four groups are not significantly different, and the conclusion is reached that tissue reactions were increased by a factor of approximately 1·7 by irradiation in high pressure oxygen. Photographs of mice shown in Fig. 7 demonstrate the difference in tumour response and tissue reactions in the legs of mice irradiated in either air or OHP.

*Polarographic measurements of tumour and tissue oxygen tension during OHP*

To determine the relative effect of OHP on tumour and normal tissues, mice with large Ehrlich's tumours (inoculated limbs measuring 8–9 cm. in circumference) were lightly anaesthetised with pentobarbital sodium. The surface of the tumour was exposed through a small incision and a platinum electrode inserted to a depth of 8 mm. into the tumour. A second platinum electrode was placed beneath the skin of the thigh adjacent to the tumour ; the anode was inserted either in a subcutaneous tissue site or in the rectum. The mouse was placed in the pressure chamber and current measurements made with the galvanometer sensitivity adjusted to give a reading of one scale division for the lowest reading electrode. This sensitivity setting was kept constant for the duration of each experiment. Currents were determined for the mouse breathing air, after flushing of tank with pure oxygen, after compression to various tensions of oxygen, and during decompression. Two typical records are illustrated in Fig. 5. The range of the galvanometer scale (100 divisions) was usually too small to record the full range of oxygen currents generated in normal tissues following compression of the animals with the method employed of selecting the sensitivity for each experiment (e.g. Fig. 5*a*). Although the pair of Pt cathodes used for each animal were chosen to give approximately the same values on calibration, the current values recorded are expressed as arbitrary units and only indicate relative changes for normal tissue and tumour in each individual experiment.

Attention is drawn to the following findings :—

(i) Very large variations in the cathode current in mice breathing air were found for the same electrode inserted in different sites in either normal tissues or tumours. This occurred despite the finding that after the electrode was removed from the mouse, its calibration curve remained essentially unaltered.

(ii) Whilst the initial reading (in air) for the tumour was generally lower than that for normal tissue (Fig. 5*a*), sometimes the reverse held (Fig. 5*b*).

(iii) The responsiveness of normal tissue to a change in the partial pressure of oxygen respired, was generally much greater than that of tumour (Fig. 5*a*). The rise in tumour current usually lagged behind that of normal tissue on compression, and often only rose when the tank pressure had reached 30–45 lb. per square inch. On the other hand sometimes the reverse was found and the lag was more marked for normal tissue (Fig. 5*b*).

(iv) The cathode currents in both tumour and normal tissues were often still rising 10 minutes after 60 lb. per square inch oxygen gauge pressure was reached in the tank. Most of the rise in current sometimes developed during the maintenance of this pressure level in the tank.

(v) The maximum current values reached after compression, were almost invariably much higher for normal tissues than tumours. If electrodes were placed in the necrotic centre of a tumour, the current was almost zero with the animal breathing air, and no rise occurred after breathing pure oxygen at 60 lb. per square inch gauge pressure, for as long as 20 minutes.

(vi) After decompression, the cathode current for the tumour fell much more rapidly, than the corresponding tissue current as is seen for both examples in Fig. 5 ; the lag which occurred for the fall of current in normal tissue currents was striking.

As already stated, tumours used in these experiments were large, and both macroscopic examination of the tumour after injection of animals with lissamine

FIG. 5 (*a*, upper and *b*, lower). Cathode current changes corresponding to levels of respired oxygen tension for tumour and subcutaneous tissue in anaesthetised mice. Calibration curves for the pair of cathodes used in each experiment were the same, but the galvanometer sensitivity setting (see text) differs for measurements made in the two animals illustrated. Tip of tumour electrode inserted to a depth 8 mm. below surface of tumour. Advanced tumours approximately 8 cm. in circumference used.

green (Goldacre and Sylvén, 1959) and histological examination, showed the tumour to consist of an outer vascularised zone merging into central necrosis (Fig. 6*a*). In this outer zone most of the tumour tissue was arranged in cords, approximately 80 $\mu$ in radius, surrounding capillaries. The periphery of the cord, most distant to the central capillary, merged into necrosis (Fig. 6*b*). The electrode was generally inserted into this outer portion of the tumour consisting of scattered cords amid necrosis, as in Fig. 6*b*. The size of the electrode tip (230 $\mu$) was such as to cause considerable unavoidable contusion and disruption of tissues and must be recognised in any interpretation of the results.

### DISCUSSION

The use of a tumour homograft as a radiobiological indicator is subject to criticisms, which have been stressed on several occasions (e.g. Scott, 1958; Howard Flanders and Scott, 1960). However isogenic systems are not free from similar reservations in regard to strict immunological compatibility, the number of cells inoculated required to constitute a " critical assembly " and the capacity of the tumour stroma and blood supply to keep pace with tumour growth and nutrition. For example, the ED50 values reported for tumour " take " in isologous grafts vary considerably. For C3H mouse mammary carcinoma, Cohen and Cohen (1960) have computed the ED50 for the standard isogenic situation to be 25 cells, using implanted tumour fragments. For the C3H mouse mammary carcinoma, Révész (1958) gives ED50's ranging from $>10^4$ to $<10^7$ cells in different experiments. In the experiments of Suit, Schlachter and Andrews (1960) using C3H/Ba mammary adenocarcinoma in C3H/He mice, 15 per cent of mice did not develop tumours after inoculation with $1{\cdot}8 \times 10^6$ cells, and the ED99 was $\sim 10^7$ cells. For methyl-cholanthrene induced sarcoma in A strain mice, the ED50 was $10^3$–$10^4$ cells (Révész, 1958). For DBA lymphoma the same author reported an approximate 20 per cent tumour incidence for an inoculation of 40 cells; Hewitt (1958) using a CBA lymphatic leukaemia reported ED50's of 1·6, 1·2 and 3·5 cells for intravenous, intraperitoneal and subcutaneous routes respectively.

That strict immunological compatibility does not exist in certain " standard isogenic situations " and that this factor may influence radiosensitivity of the tumour is illustrated by the work of Cohen and Cohen (1960), using the C3H mouse mammary carcinoma isograft, for which immunological attenuation of hosts by whole body irradiation or corticosteroids, reduced the estimated ED50 from 25 to 1 cell, and increased the $LD_{50}$ radiation dose from 5660 to 7500 rads. Conversely " hyperimmunisation " of host mice, increased the ED50 to 200 cells, and reduced the $LD_{50}$ to 4800 rads.

The homologous situation with Ehrlich's ascites tumour varies somewhat with different strains of this tumour and different implantation sites. For the hyperdiploid line used in our experiments, the ED50 for intramuscular inoculation in hybrid mice was 281 ($\pm 71$) cells, and reduced to $<10$ cells by immunological approximation with whole body X-irradiation. In previous experiments, using the same tumour but non-inbred mice of C3H and A origins, the resulting ED50 was not significantly different. By comparison, Warner and James (1959) using the same tumour inoculated in penbred white mice obtained an ED50 of 850 cells for the intraperitoneal route. Donaldson and Mitchell (1959) reported tumour incidences of 100 per cent and 70 per cent for intraperitoneal and subcutaneous routes respectively, after inoculation of Swiss mice with $\sim 16$ EAT cells. Using a near-tetraploid strain of EAT in non-inbred white mice, Scott (1957) obtained an ED50 of 10–15 cells for the intraperitoneal route; the ED50 (and presumably the homograft reaction) was less for the intramuscular route, but much greater for C57 black strain mice.

In view of these observations, if host-tumour transplantation compatibility is to be based on the smallness of the critical cell number for tumour " take ", the rapidity of its growth, and an absence of spontaneous regressions, there seems little to choose between the majority of standard isogenic situations and a homologous situation after immunological attenuation such as that reported in this paper. Whole body irradiation (400–500 r) caused a substantial reduction in the homograft reaction for EAT, if given 24 hours preceding inoculation, a finding supported by that of Mazurek and Duplan (1959) using a dose 350 r, 24 hours preceding inoculation. Chronic administration of Compound 48/80 depletes endogenous tissue histamine (Feldberg and Talesnik, 1953) and also other amines (Lewis, 1958), and was previously found to reduce the heterograft reaction against EAT in rats (van den Brenk and Upfill, 1958). However, no significant reduction in the homograft reaction resulted. This treatment might have been expected to interfere with the immunity reaction in so far as histamine release is generally considered to play a role in antigen-antibody reactions in tissues.

The lethal effect of irradiation on EAT cells *in vivo*, was apparently greatly reduced if whole body irradiated recipient mice were used. Immunological attenuation resulted in an apparent dose reduction by a factor of 3·5, compared with a factor of 1·3 obtained by Cohen and Cohen (1960) for C3H mouse mammary carcinoma isografts. It follows that an overriding influence on radiosensitivity of this order must be reduced to a minimum if other factors affecting radiosensitivity, such as tissue oxygen tension, are to be assessed. In experiments designed to determine the " oxygen effect " the criticism may be raised that local irradiation of the tumours was performed 7–8 days after whole body irradiation of hosts, by which time immunological recovery may have taken place. However, delay of the irradiation-inoculation interval to 7 days was found to increase the $ED_{50}$ to 31 ($\pm$17) EAT cells, which was still considerably less than that for unirradiated hosts. Also, during local irradiation of the tumours, mice received a further whole body dose of X-rays averaging 200 r (see Methods). Nossal and Larkin (1958) have shown that the immunity response remains depressed for at least 9 days in rats after sublethal irradiation with 500 r X-rays.

---

EXPLANATION OF PLATE.

FIG. 6 (*a*).—Macroscopic appearance of section of untreated solid Ehrlich's tumour removed from thigh and stained with haematoxylin-eosin. Darker areas are viable tumour, pale areas tumour necrosis.

(*b*) Microscopic appearance ($\times$ 120) of cords of viable tumour cells surrounding capillaries and merging into necrotic tumour. Pyknotic and fragmented tumour cells seen at periphery of cord. Field is selected from central portion of the tumour section in (*a*) and represents the position of a platinum electrode tip in recording changes in oxygen tension (see text).

FIG. 7.—Appearance of tumours in mice treated in air (*a, c, e*) or in 45 lb. per square inch pressure pure oxygen OHP (*b, d, f*). In each mouse tumour (T) situated in right thigh.

(*a, b*) Four weeks after 1000 r X-rays, showing much larger size of residual tumours in air treated mice (*a*), than in OHP treated mice (*b*).

(*c, d*) Four weeks after 2000 r X-rays. No residual tumour seen in 3 OHP treated mice (*d*), but residual tumour in the two of 3 air treated mice on right (*c*). Skin reactions are much more severe in OHP treated mice, the treated limbs showing severe moist desquamation and retraction.

(*e, f*) Same 6 mice (2000 r) as (*c, d*) but six weeks later. Showing cure of tumours in OHP treated mice (*f*); tissue reactions have subsided and epilation of treated limbs is seen. Two air treated mice on right in (*e*) have large residual tumours invading tissues of abdomen and causing oedema of foot; remaining mouse on left shows no residual tumour; the dose of irradiation (2000 r) has not caused epilation.

6a

6b

7

van den Brenk.

The studies reported for growth rate of solid tumours resulting from cell inocula of different orders, demonstrated the unreliability of this method in determining the number of cells remaining after a sublethal treatment. The total number of cells which have accumulated at any one time in a solid tumour is largely an index of *tissue form* and a result of the complex interactions of similar and dissimilar cells which go to make up an " organised " tumour. In solid Ehrlich's tumour, after 7–10 days of growth, the major portion of the tumour is necrotic, and the stroma and vasculature is limited in its capacity to meet the nutritional requirements and becomes a major factor in determining the tumour size and its viability. In assessing the radiation doses needed to cope with " small " tumours and " large " tumours, many investigators have made calculations based on the data derived from tissue culture studies. Employing more and more artificial conditions of growth *in vitro*, radiosensitivity curves have been obtained, which may be wrongly interpreted in relation to growth and radiosensitivity *in vivo*. So-called " large " tumours *in vivo*, being largely necrotic, may contain many fewer cells, than estimated from their diameters or weights. Furthermore only a proportion of tumour cells may undergo division *in vivo* ; differentiation or natural senescence affects the remainder, in addition to death attributable to endogenous or exogenous factors, which interfere with nutrition. It follows that tumour size, is unsuitable to gauge any one factor affecting radiosensitivity as demonstrated by our own results.

The curves obtained for EAT irradiated *in vivo* in air and pure oxygen (45 lb. per square inch gauge pressure) clearly show an increase in radiosensitivity in OHP with a reduction in the $LD_{50}$ from 4000 r (air) to 1670 r (OHP) i.e. an " oxygen effect " factor of 2·4 for this particular situation. The close correlation of the value of the factor obtained with that of 2·3 for EAT cells irradiated *in vitro* under either complete anoxic or oxygenated conditions (Deschner and Gray, 1959) was surprising, and suggests that the tumours were anoxic during irradiation in air, whilst OHP provided full oxygenation. This suggestion is supported by the finding that OHP also caused a very marked increase in skin reactions (factorial increases ranging from 1·6–2·2).

During the first seven days after inoculation, the limb circumference ($C$) increased from approximately 2 cm. to 3 cm., and assuming the volume to be spherical in shape, this represents an increase in volume ($\delta v$) given by

$$\delta v = \frac{C_1^3 - C_2^3}{6\,\pi^2} \quad . \quad . \quad . \quad . \quad . \quad \text{(i)}$$

$$= 32 \text{ mm.}^3$$

If a tumour cell be considered to occupy an *effective* volume of $10^{-8}$ cm.$^3$ *in vivo* after allowing for the volume occupied by stroma (Cohen and Cohen, 1960), and assuming that at the time of irradiation all the cells were viable, the volume $\delta v = 32$ mm.$^3$ represents an initial tumour cell population ($n_0$) of $0{\cdot}32 \times 10^8$ cells. The inactivation of tumour cells has been shown to be exponential in the case of HeLa cells *in vitro* (Puck and Marcus, 1956) and CBA mouse lymphatic leukaemia *in vivo* (Hewitt and Wilson, 1959*a*). The relationship is given by

$$\log_e \left(\frac{n}{n_0}\right) = -\frac{D}{D_0} \quad . \quad . \quad . \quad . \quad . \quad \text{(ii)}$$

where $n$ is the number of cells surviving dose $D$ and $D_0$ is the mean lethal dose (Lea, 1955). For an $LD_{50}$ for EAT of 1670 r in high pressure oxygen and a value of $n = 31$ ($\pm 17$) cells obtained for titration in mice 7 days after sublethal whole body irradiation, substitution in equation (ii) gives a value $D_0 = 126$ r (OHP), the corresponding value for mice irradiated in air being $D_0 = 290$ r (air). This value for the inactivation dose in oxygen agrees substantially with that obtained by Puck and Marcus (1956) for HeLa cells *in vitro* ($D_0 = 96$ r ; recently adjusted by Morkovin and Feldman (1959) to $D_0 = 139$ rads owing to an error in the original dosimetry) ; by Elkind (1960) for Chinese hamster ovarian tissue *in vitro* ($D_0 = 128$ rads) and by Hewitt and Wilson (1959*a*, 1959*b*) for CBA mouse leukaemia *in vivo* ($D_0 = 162$ r for $^{60}$Co $\gamma$-rays). It is considered that this calculated value for $D_0$ in high pressure oxygen, suggests that in animals anaesthetised with pentobarbital sodium and respiring air at atmospheric pressure, the major portion of the tumour was anoxic during irradiation, despite quite elaborate attempts being made to avoid interference with blood supply of the leg, and the evidence provided by polarography and injection of dye. The very marked increase in skin reactions under OHP supports this conclusion. It is considered unlikely that the anaesthetic alone was responsible for this state of anoxia, in both tumour and skin of animals breathing air. Other investigators who have used animal tumours to determine radiocurability report $LD_{50}$ doses in unanaesthetised animals respiring air, which also seem very high. In C3H mouse mammary carcinoma, Cohen and Cohen (1960) calculated an $LD_{50}$ of 5660 rads for the standard isogenic situation ; Suit *et al.* (1960) report a value of 5500 r for the same tumour but could only demonstrate a slight effect of OHP at 30 lb. per square inch gauge pressure on cure rate. However, the tissue reactions reported were not significantly increased, and the method used to immobilise animals strongly suggests that anoxic conditions may have prevailed during irradiation which were not overcome by OHP treatment at 30 lb. pressure. Scott (personal communication) has emphasised the marked influence of apparently minor degrees of vascular compression and stasis on oxygenation of tissues. This was also stressed in the report of Gray (1957). It seems quite clear that local irradiation of animal tumours carried out by retracting a subcutaneous skin tumour away from the body causes severe tumour anoxia. For small animals further factors operate which predispose to tissue anoxia. In mice, oxygen consumption per unit body weight is very high, and the slope of the oxygen saturation curve for mouse blood is very flat in comparison with larger animals (Schmidt-Neilsen and Larimer, 1958). These factors predispose to anoxia, a higher diffusion head being required to adequately oxygenate tissues and particularly those tissues with a high oxygen consumption. Also of importance is the finding of Chase and Hunt (1959) that oxygen diffusing through the surface of skin, may modify radiosensitivity. They found that this applied particularly to damage to the superficial cutaneous appendages (resting hair follicles) and for mice respiring air, damage was reduced by nitrogen flowing over the surface of skin during irradiation. Furthermore, if the flow of blood to the skin was prevented by a vascular clamp to decrease the radiation damage to resting follicles, this effect was reversed if the animal was irradiated in a chamber under 2 atmospheres absolute pressure of pure oxygen and radiosensitivity was restored. Chase and Hunt (1959) also showed that deep barbiturate anaesthesia did not significantly affect hair follicle damage, but they mentioned that " the degree of skin damage is noticeably reduced ". As a result of polarographic measurements of oxygen tension in skin of rats in relation to radioprotective chemicals and the effect of OHP on this action (Jamieson and van den Brenk, 1960), we have found that barbiturate anaesthesia does cause some reduction in oxygen tension but that respiration of oxygen at 1 atmosphere absolute and higher, readily overcomes any skin anoxia attributable to barbiturates. Tissue anoxia caused by a potent chemical protective agent such as 5-hydroxytryptamine, and even chlorpromazine, is much more resistant in this respect. These findings were confirmed by estimations of arterio-venous oxygen saturations using Van Slyke manometry. Taking all these factors into account, together with other evidence that pure oxygen at 1 atmosphere enhances the skin damage in mice (Wilson, 1959) and that 40 lb. per square inch pressure of pure oxygen causes a marked increase in skin reactions in anaesthetised rats (Moss and Haddy, 1960), it is concluded that the skin and tissues of limbs of mice under experimental conditions is poorly oxygenated to a degree that the radiosensitivity is well below the aerobic level and indeed approaches an anaerobic value. Furthermore, the most striking evidence which supports this conclusion is to be found in the literature of reports of radiation doses given to mouse skin ranging from 4500–8500 r (single application)—doses which clearly exceed tolerance in aerobic conditions. In our own experiments we have found single doses of 4000 r unreliable in its effect on tumours, in so far as " radiation amputation " is not infrequently a result, and tends to exaggerate cure rates (as is seen in Fig. 3 for both air and OHP). For this reason it is considered that high doses of this order which exceed tissue tolerance give an erroneous evaluation of the effects of respired oxygen tension on radiocurability.

The EAT tumour is clearly at a disadvantage in relation to oxygenation. Histological examination shows progressive necrosis with growth, and vessels surrounded by cylinders of surviving tumour tissue (Fig. 6*b*), which measure approximately 80 $\mu$ in radius. We have little knowledge concerning the dynamics of blood flow in tumours in relation to capillary oxygen gradients, short circuits (arteriovenous anastomoses) and response to pharmacodynamic agents. Nor do we know the extent to which oxygen is consumed by the tumour *in vivo*, and the role of glycolysis as a metabolic mechanism. The oxygen saturation of venous blood leaving tumours has been reported high in comparison to normal tissues (Bierman, Kelly and Singer, 1952 ; Thomas, 1959) suggesting either the presence of arteriovenous shunts, altered oxygen diffusion characteristics or a reduced oxygen consumption due to a predominantly anaerobic form of metabolism.

The polarographic studies reported in this paper, suggest that this method is of limited value, particularly in providing absolute values of oxygen tension in tissues. However, one of the most serious objections to the use of polarography *in vivo*, is the damage caused by electrodes, however small, to tissues and the pooling of fluid and blood around the electrode tip. This, undoubtedly, explains the lag in response to respired oxygen tensions often encountered in both tumour and normal tissues. In optimal circumstances, virtually no lag occurs, but we have found this to be quite unpredictable for " acute " insertions. For " chronic " insertions (McLaurin, Nichols and Newquist, 1959) the electrode tip doubtless becomes surrounded by a pocket of granulation tissue, which would in itself have altered vascular characteristics, and fail to be representative of the respective organ, tissue or tumour. However, the polarographic studies have clearly shown, that whilst the oxygen current of tumour tissue (semi-necrotic as in Fig. 6) is lower and tends to lag as respired oxygen tensions are raised, OHP at 45–60 lb.

per square inch usually raises this current to levels considerably in excess of the value for normal tissues of an animal breathing air, but not nearly so high as that of normal tissues in the mouse breathing OHP. Furthermore, the more rapid fall of the tumour current during decompression suggests that oxygen consumption at this site is high, and the available oxygen low.

The irradiation of EAT cells in the ascitic form in unanaesthetised mice, and titration of these cells in either irradiated or non-irradiated recipient mice (Tables II and III) may be expected to provide a better model for determination of the inactivation dose ($D_0$) and the oxygen effect factor. Whilst the data provided in this paper would need to be extended considerably for accurate calculation of the inactivation dose, an approximate estimate of $D_0$ made for $10^2$ and $10^3$ cell inocula gives the following values :—

| | $n_0$ | $n$ | $D_0$ |
|---|---|---|---|
| Irradiated Recipients | $10^2$ | 10 | 2[illegible]0 |
| | $10^3$ | 10 | 310 |
| Unirradiated Recipients | $10^3$ | 250 | 240 |

It will be noted that these values agree fairly well with the value for $D_0$ of 290 r for tumours irradiated in air and for which it has been argued that radiosensitivity approached the anaerobic level. Intraperitoneal measurements of oxygen tension in mouse ascites fluid using the Hersch analyser (Gray, 1959) gave a mean value of 14·6 mm. Hg for animals breathing air, and this value corresponded to a radiosensitivity only 20 per cent higher than the anaerobic value, for radiosensitivity of EAT cells determined *in vitro*. Gray also showed that, by administration of pure oxygen at atmospheric pressure, the radiosensitivity was still only 60 per cent above the anaerobic value. This evidence gives support to the calculations made above and the interpretation that radiosensitivity was determined at approximately anaerobic levels for tumour ascites fluid in unanaesthetised mice breathing air.

### SUMMARY

The effect of breathing high pressure oxygen (OHP) at 45 lb. per square inch gauge pressure on radiosensitivity of Ehrlich's tumour (solid form) *in vivo* has been studied. Recipient mice were given 400 r whole body irradiation before inoculation of tumour cells to cause " immunological approximation " and a substantial reduction in the homograft reaction. The effect of irradiation was based on cure of tumours 8 weeks after irradiation. Regression curves show that OHP caused a significant increase in radiosensitivity (radiocurability) with a dosage reduction by a factor of 2·4 ($\pm$0·5 SE). Tissue reactions assessed 4 weeks after irradiation were increased in OHP by factors ranging from 1·6 to 2·2. Evidence is provided that in mice breathing air at atmospheric pressure, tissue radiosensitivity approached levels corresponding to minimum (anaerobic) values. The results of polarographic measurements of oxygen tension in normal tissues and tumours in animals breathing air and OHP are described and discussed.

I am grateful to Dr. O. C. A. Scott, British Empire Cancer Campaign Radiobiology Unit, Mount Vernon Hospital, England, for his advice and criticism expressed in our correspondence over the past two years. I am indebted to Miss S. Thomas of this Unit for help with statistical analysis and thank Dr. J. Martin and his Staff in the Physics Department of this Institute for radiation dose measurements. I thank my own technical staff, in particular Mrs. K. Elliott, Miss H. Hutchings and Mr. A. Rowlatt for excellent assistance and Mrs. I. Tamosaitis for secretarial assistance. I am indebted to my brother, Mr. A. H. van den Brenk for the design and construction of high pressure oxygen equipment.

Compound 48/80 was kindly supplied by the Wellcome Research Foundation, London.

### REFERENCES

BEAN, J. W.—(1945) *Physiol. Rev.*, **25**, 1.

BIERMAN, H. R., KELLY, K. H. AND SINGER, G.—(1952) *J. nat. Cancer Inst.* **12**, 701.

VAN DEN BRENK, H. A. S. AND UPFILL, J.—(1958) *Aust. J. Sci.*, **21**, 20.

CHASE, H. B. AND HUNT J. W.—(1959) 'Pigment Cell Biology'. New York (Academic Press Inc.), p. 537.

CHURCHILL-DAVIDSON, I., SANGER, C. AND THOMLINSON, R. H.—(1957) *Brit. J, Radiol.*, **30**, 406.

COHEN, A. AND COHEN, L.—(1960) *Nature, Lond.*, **185**, 262.

DAVIES, P. W. AND BRINK, F.—(1942) *Rev. sci. Instrum.*, **13**, 524.

DESCHNER, E. E. AND GRAY, L. H.—(1959) *Radiation Res.*, **11**, 115.

DITTRICH, W. AND STUHLMAN, H.—(1954) *Naturwissenschaften*, **41**, 122.

DONALDSON, D. M. AND MITCHELL, J. R.—(1959) *Proc. Soc. exp. Biol. N.Y.*, **101**, 204.

ELKIND, M. M.—(1960) *Radiology*, **74**, 529.

FELDBERG, W. AND TALESNIK, J.—(1953) *J. Physiol.*, **120**, 550.

FINNEY, D. J.—(1952) 'Probit Analysis', 2nd Edition. London (Cambridge University Press).

GOLDACRE, R. J. AND SYLVÉN, B.—(1959) *Nature, Lond.*, **185**, 63.

GRAY, L. H.—(1957) *Brit. J. Radiol.*, **30**, 403.—(1958) 'Organic Peroxides in Radiobiology'. London (Pergamon Press).—(1959) 'Radiation Biology' ed. J. H. Martin. London (Butterworths Scientific Publications), p. 76.

*Idem*, CONGER, A. D., EBERT, M., HORNSEY, S. AND SCOTT, O. C. A.—(1953) *Brit. J. Radiol.*, **30**, 406.

HARRIS, H. AND BARCLAY, W. R.—(1955) *Brit. J. exp. Path.*, **36**, 592.

HEWITT, H. B.—(1958) *Brit. J. Cancer.*, **12**, 378.

*Idem* AND WILSON, C. W.—(1959*a*) *Ibid.*, **13**, 69.—(1959*b*) *Nature, Lond.*, **183**, 1060.

HOLLCROFT, J., LORENZ, E. AND MATHEWS, M.—(1952) *J. nat. Cancer Inst.*, **12**, 751.

HOSKINS, J. M., MEYNELL, G. G. AND SANDERS, F. K.—(1956) *Exp. Cell Res.*, **11**, 297.

HOWARD FLANDERS, P. AND SCOTT, O. C. A.—(1960) *Radiology*, **74**, 956.

JAMIESON, D. AND VAN DEN BRENK, H. A. S.—(1960) *Proc. 3rd Aust. Conf. Radiobiol.* (In the press.)

LEA, D. E.—(1955) 'Actions of Radiation on Living Cells', 2nd Edition. London (Cambridge University Press).

LEWIS, G. P.—(1958) '5-Hydroxytryptamine'. London (Symposium Publications Division, Pergamon Press), p. 26.

MCLAURIN, R. L., NICHOLS, J. B. AND NEWQUIST, R. E.—(1959) *J. appl. Physiol.*, **14**, 480.

MAZUREK, C. AND DUPLAN, J. F.—(1959) *Bull. Ass. franç. Cancer*, **46**, 119.

MORKOVIN, D. AND FELDMAN, A.—(1959) *Brit. J. Radiol.*, **32**, 282.

MOSS, W. T. AND HADDY, F. J.—(1960) *Radiology*, **75**, 55.

NOSSAL, G. J. V. AND LARKIN, L.—(1958) 'Radiation Biology,' ed. J. H. Martin. London (Butterworths Scientific Publications), p. 236.

PUCK, T. T. AND MARCUS, P. I.—(1956) *J. exp. Med.*, **103**, 653.

Révész, L.—(1958) *J. nat. Cancer Inst.*, **20**, 1157.
du Salt, L. A., Eyler, W. R. and Dobben, D.—(1959) *Amer. J. Roentgenol.*, **82**, 688.
Schmidt-Neilsen, K. and Larimer, J. L.—(1958) *Amer. J. Physiol.*, **195**, 424.
Scott, O. C. A—(1957) *Brit. J. Cancer*, **11**, 130.—(1958) ' Advances in Biological and Medical Physics '. New York (Academic Press), Vol. **6**, 121.
Suit, H., Schlachter, L. and Andrews, J. R.—(1960) *J. nat. Cancer Inst.*, **24**, 1271.
Thomas, J.—(1959) *Z. Geburtsh. Gynäk.*, **152**, 113, 291.
Tödt, F., Teske, G., Windisch, F., Heumann, W. and Goslich, C.—(1952) *Biochem. Z.*, **323**, 192.
Warner, P. and James, A. T.—(1959) *Brit. J. Cancer*, **13**, 288.
Wilson, C. W.—(1959) *Brit. J. Radiol.*, **32**, 383.